# Beyond structures: solution NMR as the quantitative engine of integrated structural biology

**DOI:** 10.3389/fmolb.2026.1941622

**Published:** 2026-09-11

**Authors:** Jeffrey A. Purslow, Vincenzo Venditti

**Affiliations:** 1 Roy J. Carver Department of Biochemistry, Biophysics and Molecular Biology, Iowa State University, Ames, IA, United States; 2 Department of Chemistry, Iowa State University, Ames, IA, United States

**Keywords:** allostery, biomolecular dynamics, biomolecular mechanisms, integrated structural biology, solution NMR

## Abstract

Structural biology is moving beyond the determination of static molecular structures toward quantitative descriptions of biomolecular mechanisms. Modern questions increasingly focus on conformational heterogeneity, exchange kinetics, weak and transient interactions, allostery, disorder, assembly, and phase behavior. These properties are often difficult to infer from crystallography, cryo-electron microscopy, scattering, imaging, mass spectrometry, molecular simulations, or structural prediction alone. Solution NMR spectroscopy occupies a distinctive position in this integrated landscape because it provides residue- and atom-specific observables that report on structure, dynamics, populations, kinetics, and interactions directly in solution. Here, we review the role of solution NMR in integrated structural biology from a question-centered perspective. Rather than organizing the discussion around individual NMR experiments, we ask how NMR can be used with complementary approaches to address recurring mechanistic problems: What is the structure of a biomolecule in solution? What conformational states are populated, and how rapidly do they interconvert? How do ligands, nucleic acids, membranes, surfaces, or partner proteins bind? How are allosteric signals transmitted? How do disorder, large assemblies, and biomolecular condensates regulate function? How can NMR observables be integrated with simulations and other structural restraints to build testable models? The future of solution NMR lies not primarily in isolated structure determination, but in its ability to transform structural models into dynamic, quantitative, and experimentally constrained descriptions of biomolecular mechanism.

## 1 Introduction: structural biology after the single-structure era

The central goal of structural biology has expanded. For much of the field’s history, the determination of a high-resolution structure represented a natural endpoint. Atomic structures of proteins, nucleic acids, and macromolecular complexes transformed biology by revealing active sites, binding pockets, recognition interfaces, catalytic architectures, and the molecular logic of biological specificity (Ward et al., 2013). X-ray crystallography provided the first atomic views of many biomolecules (Perutz et al., 1960; Kendrew et al., 1958). Cryo-electron microscopy (cryo-EM) has now made it possible to visualize large macromolecular assemblies, membrane proteins, molecular machines, and conformational states that were previously inaccessible (Kuhlbrandt, 2014; Bai et al., 2015). Computational structure prediction has further altered the landscape by providing high-quality structural models for vast regions of protein sequence space (Jumper et al., 2021). These developments have been transformative.

Yet many mechanistic questions cannot be answered by a single structure, regardless of its resolution. Biomolecules function in solution, in crowded environments, at interfaces, in assemblies, and often far from a single rigid conformation. Enzymes fluctuate among states that differ in catalytic competence (Henzler-Wildman and Kern, 2007; Burns et al., 2024; Dotas et al., 2020; Boehr et al., 2006; Eisenmesser et al., 2002; Eisenmesser et al., 2005; Nguyen et al., 2018). Receptors and signaling proteins respond to ligands by shifting pre-existing conformational equilibria (Cooper and Dryden, 1984; Tang et al., 2007; Venditti and Clore, 2012; Venditti et al., 2015a). Weak and transient interactions can determine specificity even when they are difficult to trap structurally (Dyson and Wright, 2005; Fawzi et al., 2010; Iwahara and Clore, 2006; Tang et al., 2006). Intrinsically disordered regions mediate regulation through dynamic ensembles rather than stable folds (Hyman et al., 2014; Adamski et al., 2019; Kriwacki et al., 1996). Biomolecular condensates depend on networks of weak, multivalent, and time-dependent contacts (Burke et al., 2015; Rizuan et al., 2025). Allosteric regulation can arise from changes in conformational populations, fluctuations, entropy, and kinetic barriers, even when average structures change only subtly (Cooper and Dryden, 1984; Putnam et al., 2007; Popovych et al., 2009; Tzeng and Kalodimos, 2009; Tzeng and Kalodimos, 2012).

These problems have driven the rise of integrated structural biology. In this framework, no single method is expected to provide a complete molecular description. High-resolution structures from crystallography or cryo-EM are combined with solution scattering, small-angle neutron scattering (SANS), crosslinking mass spectrometry (XL-MS), hydrogen-deuterium exchange mass spectrometry (HDX-MS), electron paramagnetic resonance (EPR), fluorescence spectroscopy, microscopy, biochemical assays, molecular simulations, and increasingly machine-learning-based structural models. Each method contributes a different projection of the molecular system. The challenge is to combine these projections into a coherent, quantitative, and experimentally testable model.

Solution NMR spectroscopy occupies a distinctive position in this ecosystem. It is sometimes described narrowly as a method for determining structures of small proteins in solution. That view is historically accurate but conceptually incomplete. In modern structural biology, solution NMR is most powerful not when it is asked simply to produce a static structure, but when it is used to quantify molecular behavior that is invisible, averaged, or underdetermined by other methods. NMR reports on atomic and residue-specific environments, conformational exchange, dynamics over a broad range of timescales, weak binding, transient encounter complexes, low-population excited states, ligand-dependent equilibria, and coupled changes in structure and motion (Venditti and Clore, 2012; Venditti et al., 2015a; Iwahara et al., 2004; Kay, 1998; Palmer, 2004; Palmer, 2015; Wuthrich, 2001; Venditti et al., 2008; Venditti et al., 2009). It can detect states that are too sparsely populated to dominate a cryo-EM reconstruction (Huang et al., 2023), too transient to crystallize (Bouvignies et al., 2011), too dynamic to define by a single model (Sedinkin et al., 2023; Sedinkin et al., 2024), or too heterogeneous to describe with one structural snapshot (Purslow et al., 2018; Purslow et al., 2021).

This review is organized around questions rather than methods. We do not begin by asking what each NMR experiment measures. Instead, we ask what a structural biologist or biophysicist might want to know about a biomolecular system, and then discuss how solution NMR can contribute to the answer in combination with other approaches. This organization reflects a practical reality: most biological projects do not start with a pulse sequence. They start with a mechanistic question. Does a protein adopt the same structure in solution as in a crystal? Does a ligand select a pre-existing conformation or induce a new one? Which residues form a transient protein-protein interface? Does a disordered region contain residual structure? Does allostery involve a structural pathway, a redistribution of dynamics, or a shift in populations? Does a condensate contain specific molecular contacts, or is it governed by nonspecific polymer-like interactions? Which conformational state is functional?

This question-centered Review is intended for readers who are not necessarily specialists in solution NMR, including structural biologists, molecular biologists, biochemists, and researchers encountering the field for the first time. Our goal is not to provide an exhaustive methodological review or a detailed experimental manual. Instead, we aim to organize solution NMR around the biological questions it can help answer, and to clarify how different classes of NMR observables can be integrated with complementary structural, computational, and functional approaches. The examples discussed are therefore selective rather than comprehensive, and the boundaries between experimental regimes are presented as practical guides rather than strict classifications.

**FIGURE 1:**
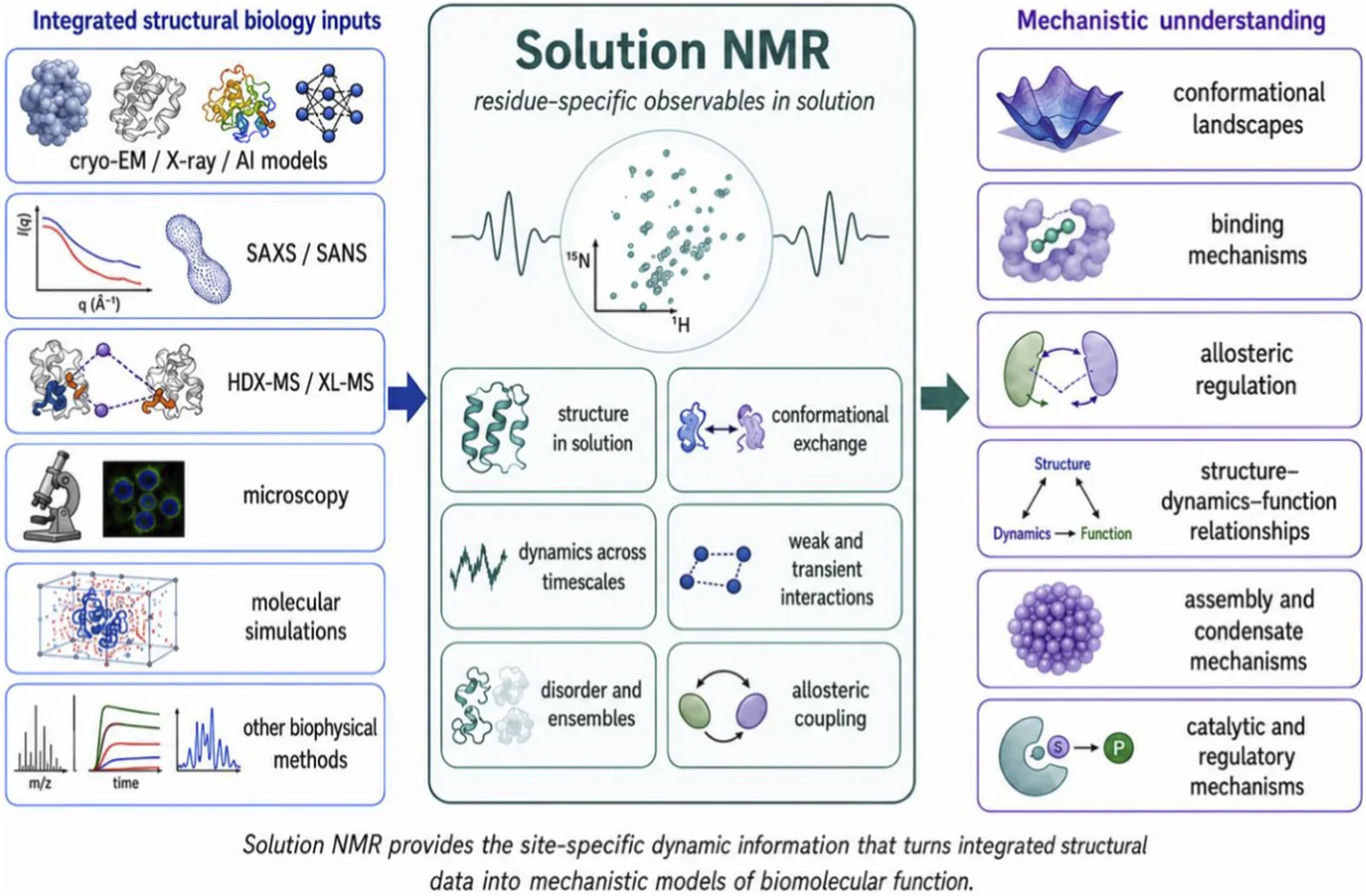
Solution NMR as the bridge from structure to mechanism. Integrated structural biology provides structural models, global constraints, simulations, imaging, and other biophysical measurements. Solution NMR adds residue-specific information on structure, dynamics, exchange, weak interactions, disorder, and allosteric coupling in solution, thereby converting structural descriptions into mechanistic models of biomolecular function.

By mapping NMR approaches onto these questions, we aim to highlight solution NMR as one quantitative component of integrated structural biology: a method that connects structures to ensembles, ensembles to kinetics, kinetics to thermodynamics, and molecular properties to biological function. This role of solution NMR as a bridge between integrated structural biology inputs and mechanistic understanding is summarized in Figure 1.

## 2 From method-centered NMR to question-centered NMR

NMR spectroscopy is methodologically rich. A traditional review might be organized around chemical shifts, scalar couplings, nuclear Overhauser effects (NOEs), residual dipolar couplings (RDCs), paramagnetic relaxation enhancements (PREs), pseudocontact shifts (PCSs), relaxation measurements, relaxation dispersion, chemical exchange saturation transfer (CEST), dark-state exchange saturation transfer (DEST), diffusion, hydrogen-exchange measurements, methyl transverse relaxation-optimized spectroscopy (methyl-TROSY), and fluorine NMR (Iwahara and Clore, 2006; Wuthrich, 2001; Bax, 2003; Cavanagh et al., 2007; Gronenborn, 2022; Mittermaier and Kay, 2006; Tugarinov et al., 2004; Vallurupalli et al., 2017; An et al., 2022; Bertini et al., 2005; Venditti et al., 2016; Venditti and Fawzi, 2018; Bernini et al., 2009). Such organization is valuable for specialists, but it can obscure the broader logic of experimental design. To non-specialists, the diversity of NMR experiments may appear as an intimidating catalog of techniques rather than a coherent set of tools for answering molecular questions.

A question-centered framework reverses the logic. The starting point is not the experiment but the unknown. The central principle is that the biological uncertainty should determine the NMR observables, not the other way around. If the unknown is the solution structure, the relevant NMR observables are those that restrain local geometry, long-range distances, and domain orientations. If the unknown is conformational heterogeneity, the relevant observables are those that report on exchange, populations, and state-specific environments. If the unknown is binding, the relevant measurements depend on the affinity, exchange regime, molecular size, and expected lifetime of the complex. If the unknown is allostery, the central challenge is to determine how perturbations at one site alter structure, dynamics, and energetics at distant sites. If the unknown is phase separation, the task is to relate residue-level contacts and motions to mesoscale material properties.

This framing also clarifies the relationship between NMR and other structural biology methods. Cryo-EM and crystallography can define structural endpoints or dominant conformational states (Shoemaker and Ando, 2018; Venien-Bryan et al., 2017). Small-angle X-ray and neutron scattering (SAXS and SANS) report on global molecular dimensions, shape, and conformational heterogeneity in solution (Chavas, 2014; Svergun, 2010). Hydrogen-deuterium exchange mass spectrometry (HDX-MS) reports on regional backbone protection and solvent accessibility (Konermann et al., 2011; Mayne, 2016), whereas single-molecule Förster resonance energy transfer (smFRET) provides distance distributions and conformational transitions at the single-molecule level (Ha et al., 1996; Okamoto et al., 2018). Crosslinking and native mass spectrometry can provide proximity restraints (Leitner et al., 2020; O'Reilly and Rappsilber, 2018), stoichiometry, and composition (Barth and Schmidt, 2020), while microscopy defines cellular localization, assembly morphology, and phase behavior (Lippincott-Schwartz et al., 2001; Shin and Brangwynne, 2017; Zweckstetter, 2021). Molecular dynamics (MD) simulations can propose atomic mechanisms and generate structural ensembles (Karplus and McCammon, 2002; Shaw et al., 2010). NMR complements these approaches by providing solution-state, site-specific information on structure, dynamics, exchange, and interactions that can be used to test, refine, and interpret the resulting models.

**FIGURE 2:**
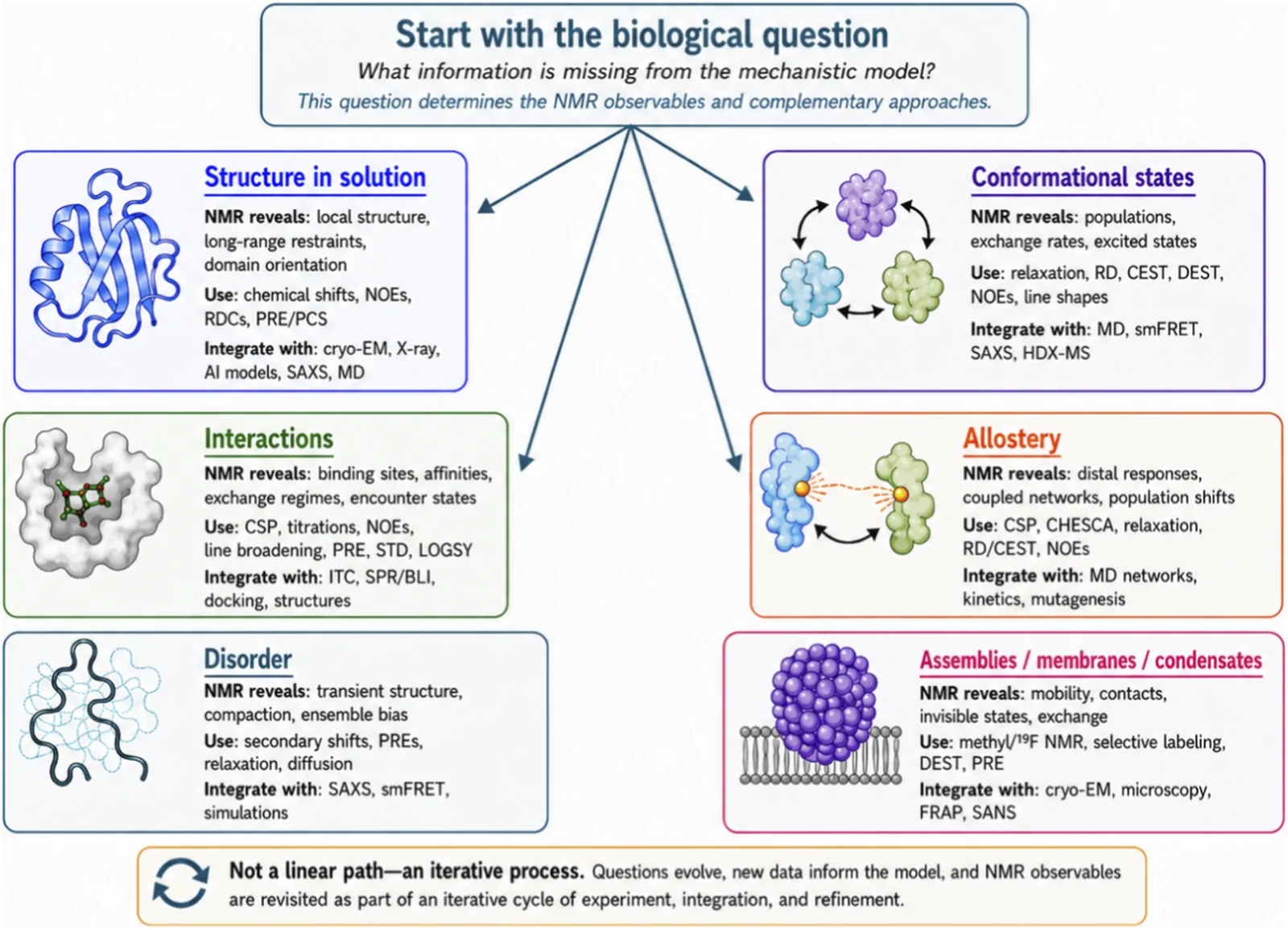
A question-centered framework for selecting solution NMR strategies. The choice of solution NMR experiments should be driven by the biological question being asked. Different questions (ranging from structure in solution and conformational exchange to interactions, allostery, disorder, and complex assemblies) require different NMR observables and are most powerful when integrated with complementary structural, computational, and functional approaches. A more exhaustive list of experiments is listed in Table 2.

The sections below are therefore organized by biological and biophysical questions. For each question, we emphasize three points: what NMR can uniquely contribute, which NMR observables are most relevant, and how those observables are most productively integrated with complementary methods. This logic is summarized in Figure 2, which maps common biological questions onto the NMR observables and complementary approaches most useful for addressing them. For readers less familiar with these methods, Table 1 provides a concise overview of selected NMR and complementary approaches, their commonly used abbreviations, the information they report, and the biological questions they can help address.

**TABLE 1:** Selected NMR and complementary methods used in the question-centered framework.

Method	What it reports	Typical biological question
Residual dipolar coupling (RDC)	Average bond-vector orientations in weakly aligned samples	Are secondary-structure or domain orientations consistent with the solution-state model?
Paramagnetic relaxation enhancement (PRE)	Distance-weighted proximity to an introduced paramagnetic center	Are transient, long-range, or low-population contacts present?
Pseudocontact shift (PCS)	Long-range positional and orientational information relative to a paramagnetic center	How are domains or ligands positioned relative to one another?
Carr–Purcell–Meiboom–Gill (CPMG)/rotating-frame (R_1ρ_) relaxation dispersion	Exchange rates, populations, and chemical-shift differences for interconverting states	Does the molecule transiently populate a functionally relevant alternative conformation?
Chemical exchange saturation transfer (CEST)	Exchange with a low-population state detected by saturation transfer	Are sparsely populated conformations present and exchanging with the major state?
Dark-state exchange saturation transfer (DEST)	Exchange with broad or NMR-invisible high-molecular-weight states	Does an observable species transiently associate with a large assembly, surface, or condensate-associated state?
Water-ligand observed *via* gradient spectroscopy (WaterLOGSY)	Ligand binding detected through magnetization transfer involving water	Does a small molecule bind the target?
Methyl transverse relaxation-optimized spectroscopy (methyl-TROSY)/selective labeling	Site-specific probes with improved sensitivity in large biomolecules	How do selected sites in a large protein or complex respond to binding or allosteric perturbation?
Cryo-electron microscopy (cryo-EM)	Three-dimensional density maps and architectures of macromolecules and assemblies	What are the dominant structural states or overall architecture of a large assembly?
Small-angle X-ray scattering (SAXS)	Ensemble-averaged molecular dimensions and global shape in solution	Does conformational change or assembly alter the global solution architecture?
Hydrogen-deuterium exchange mass spectrometry (HDX-MS)	Regional backbone protection and solvent accessibility	Which regions change protection, flexibility, or exposure upon perturbation?
Single-molecule Förster resonance energy transfer (smFRET)	Single-molecule distance distributions and transitions between labeled sites	Are distinct global conformations or dynamic subpopulations populated?

## 3 What is the structure of the biomolecule in solution?

The most familiar application of solution NMR is structure determination. For appropriately sized proteins, nucleic acids, and complexes, NMR can provide atomic structural models using interproton distances from NOEs (Clore et al., 1998a; Wuthrich, 2003), dihedral restraints from chemical shifts and scalar couplings (Clore and Gronenborn, 1998; Cornilescu et al., 1999), orientational information from RDCs (Bax, 2003; Clore and Schwieters, 2004; Hus et al., 2001; Tjandra and Bax, 1997), and long-range restraints from paramagnetic probes (Clore et al., 2007; Iwahara et al., 2007). This classical role remains important, especially for systems that are difficult to crystallize, dynamic in solution, or too small for routine cryo-EM.

However, the role of NMR in structure determination has changed. In integrated structural biology, NMR is often used not to determine an entire structure *de novo*, but to evaluate, refine, and contextualize structures obtained by other methods. A crystal or cryo-EM structure may provide a high-resolution model of a dominant state, but NMR can determine whether that model is consistent with the solution environment. Cryo-EM provides three-dimensional density maps of vitrified macromolecules and is particularly powerful for large assemblies, whereas NMR provides local solution-state information on flexible regions and transient states that may be averaged or poorly resolved in a reconstruction (Shoemaker and Ando, 2018; Venien-Bryan et al., 2017). Chemical shifts report sensitively on local backbone and side-chain conformations (Camilloni et al., 2012; Cavalli et al., 2007; Shen et al., 2009; Wishart and Sykes, 1994). RDCs report on the average orientations of internuclear bond vectors and are therefore useful for testing secondary-structure geometry and relative domain orientations (Bax, 2003; Venditti et al., 2016). PREs exploit the strongly distance-dependent relaxation effects produced by a paramagnetic center to provide long-range proximity information and to detect transient or low-population contacts (Clore et al., 2007; Iwahara et al., 2007). PCSs provide complementary long-range positional and orientational information relative to a paramagnetic center (Pintacuda et al., 2007; Schmitz et al., 2012; Zuiderweg, 2002). Hydrogen exchange and solvent PREs can reveal protection patterns and local stability (Bernini et al., 2009; Long et al., 2014). Together, these data can determine whether the solution ensemble resembles the static model or whether important deviations occur.

This distinction is especially important for multidomain proteins, flexible complexes, and systems containing disordered regions. A high-resolution structure may describe individual domains accurately while leaving their relative orientations poorly defined. In such cases, RDCs (Tjandra and Bax, 1997; Esteban-Martin et al., 2010), PREs (Iwahara et al., 2004), PCS data (Schmitz et al., 2012; Saio et al., 2010), and SAXS profiles (Svergun, 2010) can be combined with rigid-body modeling or molecular simulations to generate solution ensembles (Venditti et al., 2016; Venditti et al., 2015b). Similarly, regions missing from crystallographic or cryo-EM density are often not simply irrelevant, rather they may mediate regulation, binding, localization, degradation, or phase separation. NMR chemical shifts, relaxation, and PREs can characterize these regions even when they are invisible to methods that favor ordered density (Dyson and Wright, 2021).

A modern structure-centered NMR workflow therefore often begins with an external model derived from crystallography, cryo-EM, structural prediction, or molecular simulations. Experimental NMR observables can then be compared with values predicted or back-calculated from the candidate model. Chemical shifts provide residue-specific tests of local backbone and side-chain environments. RDCs can be back-calculated from structural coordinates and compared with experiment to test the relative orientations of secondary-structure elements or domains. PREs provide complementary long-range information: relaxation enhancements measured from a defined paramagnetic center can be compared with those expected from candidate structures or structural ensembles. Agreement across several independent observables increases confidence that a model captures the solution state, whereas systematic discrepancies can identify incorrect local conformations, inaccurate domain orientations, flexible regions, or alternative states that are absent from the starting model. Because NMR observables are often ensemble averaged, disagreement does not necessarily imply that a structural model is globally incorrect; rather, it may indicate that a single structure provides an incomplete description of the conformational ensemble.

A representative example is bacterial Enzyme I, for which AlphaFold-derived structural models were combined with coarse-grained simulations and solution NMR to characterize the solution ensembles of its open and closed conformations (Sedinkin et al., 2023). This type of workflow illustrates how a predicted structure can serve as a starting hypothesis rather than a final answer: experimental NMR data can test whether the proposed conformations are populated in solution and help refine the resulting ensemble. More generally, integration of NMR with cryo-EM or predicted structures is most powerful when the structural model defines the candidate architecture and NMR provides independent, solution-state observables that test the local structure, relative domain organization, dynamics, and presence of alternative conformations.

These integrated approaches nevertheless have important practical and interpretive limitations. Solution NMR remains constrained by molecular size, spectral overlap, sensitivity, and isotope-labeling requirements, while observables such as RDCs and PREs are ensemble averaged and may be compatible with more than one structural model. PRE interpretation additionally depends on the position and flexibility of the paramagnetic probe, and apparent disagreement with a structural model may reflect conformational heterogeneity rather than an incorrect structure.

For non-specialists, the key principle is that NMR is most informative when matched to the remaining structural uncertainty. If the question concerns local structure, chemical shifts and NOEs provide sensitive restraints (Clore et al., 1998a; Shen et al., 2009). If the uncertainty involves domain orientation, RDCs and PCS restraints provide orientational information (Bax, 2003; Schmitz et al., 2012). If transient or low-population contacts are suspected, PREs are particularly powerful (Clore, 2008). Methyl-specific probes extend these approaches to larger systems (Tugarinov et al., 2004). The most useful structural NMR experiments are therefore those selected according to the unresolved biological or structural question after other methods have contributed their information.

## 4 What conformational states are populated?

Many biomolecules cannot be described by a single conformation. They populate ensembles of states separated by different free-energy barriers and exchanging on timescales ranging from picoseconds to seconds or longer (Henzler-Wildman and Kern, 2007; Kay, 1998; Palmer, 2004). These states may differ subtly in side-chain packing, loop organization, domain orientation, ligand-binding competence, catalytic geometry, or interaction propensity. Often, minor states are functionally important despite being sparsely populated. They may represent excited states selected by ligands, intermediates along folding or binding pathways, autoinhibited conformations, or conformations poised for catalysis.

**FIGURE 3:**
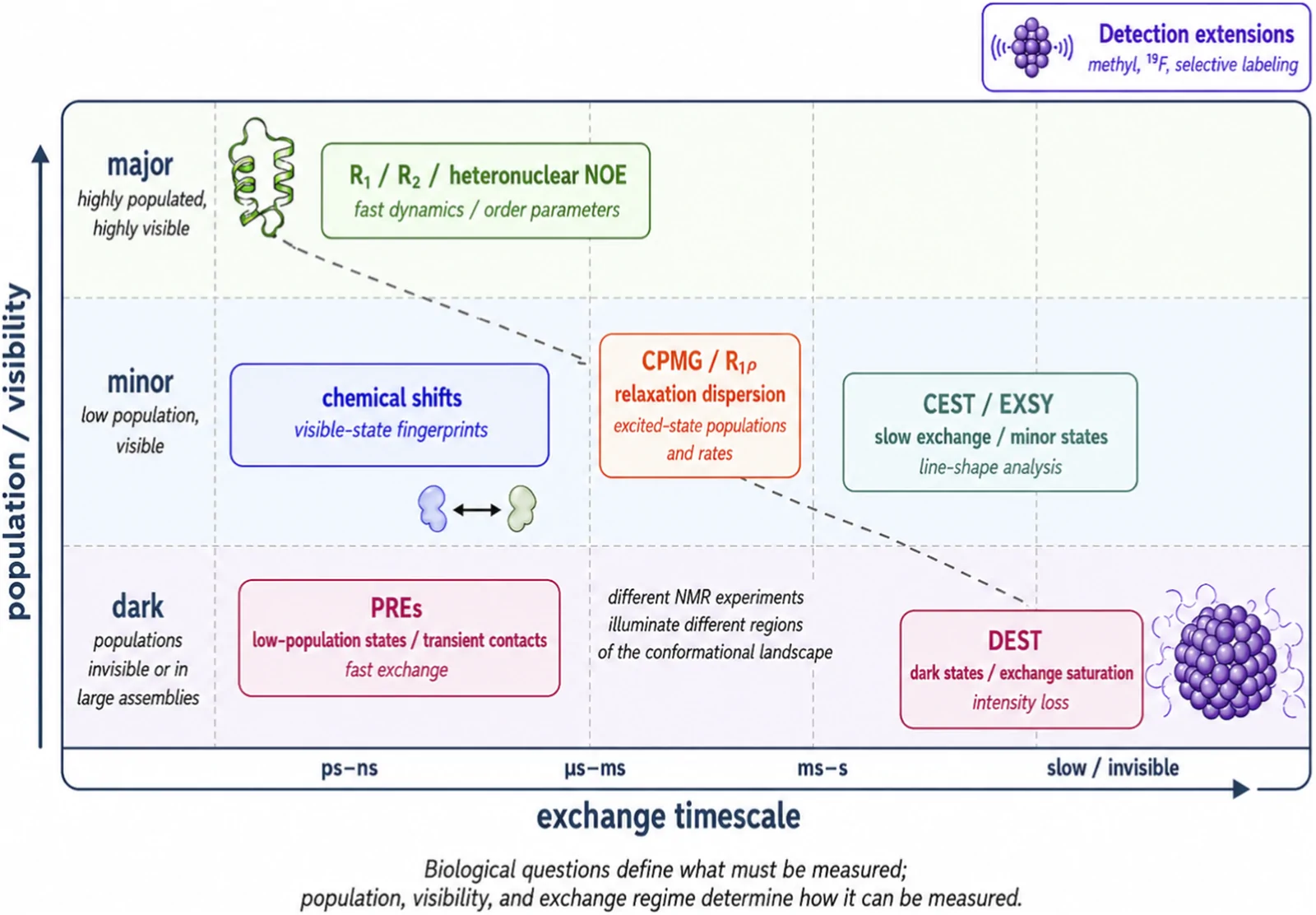
Matching NMR observables to population, visibility, and exchange regime. Solution NMR methods differ in the types of conformational states they can detect. Major, directly visible states are readily characterized by chemical shifts, relaxation measurements, NOEs, and related observables. Minor states can also be detected when they exchange with an observable state on a favorable timescale; for example, CPMG and R1ρ relaxation dispersion are powerful for μs–ms exchange, whereas CEST, EXSY, and line-shape analysis are useful for slower exchange regimes. The distinction between “visible,” “minor,” and “dark” states is therefore schematic rather than absolute: detectability depends on population, exchange rate, chemical-shift differences, linewidths, molecular size, labeling strategy, and experimental sensitivity. PREs are shown separately because they are especially sensitive to low-population transient contacts in fast exchange, where short-lived close approaches can produce measurable relaxation enhancements. DEST and related saturation-transfer approaches are most useful for states that are broadened, very large, aggregated, phase-separated, or otherwise invisible in conventional spectra. Thus, the map should be read as a practical guide to dominant regimes of applicability, not as a set of strict boundaries. Within each box, bold text denotes the principal NMR observable, regular text the information obtained, and italic text the characteristic regime or application.

This is one of the areas in which solution NMR has a unique role. Because NMR observables are sensitive to both structure and motion, they can report not only on the dominant state but also on exchange among states. The appropriate experiment depends on the population, visibility, and exchange regime of the state of interest. Major, directly visible states, low-population excited states, and dark or broadened states occupy different regions of NMR observability space and therefore require different experimental strategies (Figure 3).

Fast picosecond-to-nanosecond motions are commonly characterized using longitudinal (R_1_) and transverse (R_2_) relaxation rates, heteronuclear NOEs, and derived order parameters (Palmer, 2004; Palmer and Koss, 2019). These measurements report primarily on local internal dynamics and can reveal ligand-, mutation-, or modification-induced changes in flexibility that influence molecular recognition, catalysis, or regulation.

Microsecond-to-millisecond conformational exchange can be studied by relaxation-dispersion (RD) approaches, including Carr-Purcell-Meiboom-Gill (CPMG) and rotating-frame (R_1ρ_) experiments. These methods measure exchange contributions to transverse or rotating-frame relaxation and can provide exchange rates, populations, and chemical-shift differences between interconverting states (Palmer, 2004; Palmer and Koss, 2019; Singh et al., 2021). Biologically, they can reveal sparsely populated catalytic, ligand-competent, folding, or allosteric states that are difficult to observe directly.

For slower exchange regimes, chemical exchange saturation transfer (CEST), dark-state exchange saturation transfer (DEST), exchange spectroscopy (EXSY), and line-shape analysis provide complementary information (Palmer, 2004; An et al., 2022; Clore and Gronenborn, 1998; Anthis and Clore, 2015; Fawzi et al., 2011; Waudby et al., 2016). CEST detects low-population states through saturation transfer during exchange with an observable state, whereas DEST is particularly useful when that state exchanges with a broad or otherwise NMR-invisible species, such as a large assembly. EXSY and line-shape analysis provide additional information on exchange pathways and kinetics. These methods therefore address whether biological function depends on rare conformations or transient association with states that cannot be observed directly.

Representative studies illustrate how these measurements can be connected directly to biological mechanism. In the catabolite activator protein (CAP), NMR measurements of internal dynamics showed that changes in conformational entropy can strongly modulate DNA-binding affinity even when the binding interfaces are structurally similar, demonstrating how fast internal motions can regulate molecular recognition in ways that are not apparent from static structures (Tzeng and Kalodimos, 2012). In cyclophilin A, relaxation-dispersion measurements revealed microsecond-to-millisecond motions that are already present in the substrate-free enzyme and occur on timescales comparable to catalytic turnover, supporting a mechanistic link between pre-existing conformational dynamics and catalysis (Eisenmesser et al., 2005). At the opposite limit of NMR visibility, DEST was used to characterize exchange between soluble amyloid-β monomers and large, NMR-invisible protofibrils, providing residue-specific information on transient association with the protofibril surface (Fawzi et al., 2011). These examples illustrate how measurements of motion and exchange become mechanistically informative when the observed populations and timescales can be related to binding, catalysis, or assembly.

The power of these methods lies in their quantitative output. NMR can provide exchange rates, populations, chemical-shift differences between states, and sometimes structural information about minor conformers. This allows conformational heterogeneity to be treated not as experimental noise but as a measurable thermodynamic and kinetic property. A protein that appears as a single structure by crystallography may be revealed by NMR to exchange between active and inactive conformations. A ligand may shift a pre-existing equilibrium rather than create a new structure. A mutation may alter function by changing the population of a rare state rather than by changing the dominant structure.

Integrated approaches are especially valuable here. MD simulations can generate candidate conformational states and transition pathways (Karplus and McCammon, 2002; Shaw et al., 2010). NMR relaxation dispersion or CEST data can test whether these states are populated experimentally and on what timescale (Palmer, 2004; Karplus and McCammon, 2002; Palmer and Koss, 2019; Robustelli et al., 2012). Small-angle X-ray scattering (SAXS) reports ensemble-averaged molecular dimensions and global shape in solution, whereas single-molecule Förster resonance energy transfer (smFRET) reports distance distributions and transitions between labeled sites at the single-molecule level (Chavas, 2014; Svergun, 2010; Ha et al., 1996; Okamoto et al., 2018; Robustelli et al., 2012). Hydrogen-deuterium exchange mass spectrometry (HDX-MS) maps regional differences in backbone protection and solvent accessibility, complementing the residue-specific structural and dynamic information provided by NMR (Konermann et al., 2011; Mayne, 2016). Cryo-EM may capture some conformational endpoints (Shoemaker and Ando, 2018; Venien-Bryan et al., 2017), while NMR detects the exchange among them in solution. Together, these methods can transform a set of static structures into an energy landscape.

These capabilities are nevertheless constrained by the exchange regime and by the models used to interpret the data. No single exchange experiment spans all populations and timescales. Relaxation-dispersion and saturation-transfer data can become insensitive outside their optimal exchange regimes, and extraction of populations and rate constants depends on the kinetic model used for fitting. Moreover, detection of a minor state does not by itself establish its functional relevance, which must be tested by perturbation or complementary functional measurements.

For non-specialists, the central message is that NMR can answer questions about molecular populations and lifetimes that are difficult to obtain from static structures alone. For NMR specialists, the important experimental design issue is to match the exchange experiment to the timescale, population, and size of system. A relaxation dispersion experiment is therefore best viewed not simply as a measurement of relaxation, but as a way to ask whether an otherwise difficult-to-observe conformational state exists, how often it is visited, and how long it persists. CEST and DEST are not simply saturation-transfer experiments; they are tools for detecting weakly populated or broadened states that may dominate mechanism.

## 5 How do biomolecules interact?

Interactions are central to biomolecular function. Proteins bind ligands, substrates, inhibitors, nucleic acids, membranes, glycans, cofactors, metals, metabolites, and other proteins. These interactions can be tight and structurally well defined, but many biologically important interactions are weak, transient, multivalent, heterogeneous, or exchange among multiple binding modes. Such interactions are often difficult to capture by crystallography or cryo-EM, yet they can determine specificity, regulation, and signaling.

Solution NMR is particularly powerful for studying interactions because it can detect binding through changes in the observed molecule even when the complex is weakly populated. Chemical shift perturbation (CSP) experiments are widely used to map binding sites by monitoring changes in backbone amide, methyl, side-chain, or nucleic-acid resonances during titration (Purslow et al., 2020; Williamson, 2013; Thompson et al., 2019). Depending on the exchange regime, binding may produce smooth peak shifts, line broadening, separate peaks for free and bound states, or disappearance of resonances. These behaviors are not merely technical complications; they contain information about affinity, kinetics, and exchange. Line-shape analysis can extract association and dissociation rates (Williamson, 2013). Relaxation changes can identify regions that become more rigid or more dynamic upon binding (Williamson, 2013).

For small-molecule ligands, ligand-observed NMR methods such as saturation transfer difference (STD) (Mayer and Meyer, 1999; Nguyen and Venditti, 2020; Singh et al., 2026), water-ligand observed *via* gradient spectroscopy (WaterLOGSY) (Dalv et al., 2001), transferred NOE (Post, 2003), and fluorine NMR (Dalvit et al., 2003) can be used to identify binders, characterize binding epitopes, and screen fragments or inhibitors. WaterLOGSY detects binding through magnetization transfer involving bulk water and is particularly useful for distinguishing interacting from noninteracting small molecules in ligand and fragment screens. For protein-protein and protein-nucleic-acid interactions, protein-observed CSPs (Purslow et al., 2020), PREs (Purslow et al., 2020), RDCs (Purslow et al., 2020), intermolecular NOEs (Clore and Gronenborn, 1998), and exchange experiments (Anthis and Clore, 2015) can define binding surfaces, encounter complexes, and conformational changes coupled to association. PREs are especially useful for detecting transient encounter states because the paramagnetic effect scales strongly with the distance between the paramagnetic center and the observed nucleus and can therefore reveal low-population contacts that do not dominate the average structure (Purslow et al., 2020).

NMR is often most informative when combined with orthogonal binding and structural measurements. Isothermal titration calorimetry provides binding thermodynamics (Johnson, 2021). Surface plasmon resonance (SPR) (Tanious et al., 2008) and biolayer interferometry (BLI) (Bates et al., 2025) provide association and dissociation kinetics. Crystallography or cryo-EM can define stable bound conformations (Shoemaker and Ando, 2018; Venien-Bryan et al., 2017). SAXS can report on global complex formation (Venditti et al., 2016; Svergun, 2010). Mutagenesis can test the energetic contribution of residues identified by NMR (Huang et al., 1996; Leach et al., 2018). Molecular docking and simulations can generate binding models that are filtered by NMR restraints (Clore and Schwieters, 2003; Koukos and Bonvin, 2020; Oxenfarth et al., 2023). In this integrated workflow, NMR provides the residue-level map and exchange information needed to interpret bulk binding measurements mechanistically.

These measurements also have important interpretive limitations. Chemical shift perturbations may reflect direct contact or an indirect conformational response, line broadening can arise from exchange or nonspecific effects, and PREs depend on probe placement and can be dominated by transient low-population contacts. Ligand-observed experiments are powerful for screening but generally require orthogonal measurements to establish binding affinity, specificity, and biological relevance.

The question-centered view is particularly useful for interactions because the best NMR approach depends on the binding problem. If the question is simply whether a ligand binds, ligand-observed experiments may be sufficient. If the question is where it binds, CSP mapping is appropriate. If the question is how fast it binds and dissociates, titration line shapes or relaxation dispersion may be needed. If the question is whether encounter complexes exist, PREs are often powerful. If the question involves a large complex, methyl-TROSY, selective labeling, or fluorine probes may be required. Thus, the experiment should be selected not by habit but by the mechanistic unknown.

## 6 How is allostery transmitted?

Allostery is one of the clearest examples of why structural biology must move beyond static models. In classical terms, allostery describes regulation at a distance: binding or modification at one site changes the properties of another (Monod et al., 1965; Wodak et al., 2019). In structural terms, allostery is often represented as a pathway of conformational changes connecting two sites (Chen et al., 2022; Guo and Zhou, 2016). While such pathways can be real and important, many allosteric systems also involve shifts in conformational populations, changes in dynamics, redistribution of fluctuations, entropic contributions, and altered kinetic barriers (Astore et al., 2024; Motlagh et al., 2014; Popovych et al., 2006; Tzeng and Kalodimos, 2011). In some cases, the average structure changes only modestly while function changes dramatically.

Solution NMR is exceptionally well suited to allostery because it reports on local environments throughout a molecule and can detect both structural and dynamic responses to perturbation. CSP mapping can identify distal sites affected by ligand binding, mutation, phosphorylation, protonation, or changes in environmental conditions (Selvaratnam et al., 2011; Grutsc et al., 2016; Skeens and Lisi, 2023). Relaxation measurements can determine whether these perturbations alter fast-timescale dynamics (Frederick et al., 2007; Kern and Zuiderweg, 2003). Relaxation dispersion and CEST can reveal whether allostery involves changes in exchange between conformational states (Vallurupalli et al., 2017). Methyl-TROSY extends these approaches to larger proteins and complexes (Rosenzweig and Kay, 2014; Tugarinov et al., 2003a), where side-chain dynamics and packing interactions are central to allosteric regulation. RDCs and PREs can detect changes in domain orientation or transient long-range contacts (Venditti et al., 2016; Venditti and Fawzi, 2018; Anthis and Clore, 2015).

Covariance-based approaches provide another layer of information. Chemical shift covariance analysis (CHESCA) and related perturbation-response methods can identify groups of residues whose NMR observables respond coherently across a series of ligands, mutations, or conditions (Selvaratnam et al., 2011). Such correlations can reveal networks of coupled sites that are not obvious from a single structure. When combined with molecular simulations, contact-network analysis, elastic network models, or energy-landscape approaches, NMR perturbation data can be used to build mechanistic models of allosteric communication (Grutsc et al., 2016; Skeens and Lisi, 2023).

The value of integrated structural biology is especially apparent in allostery. Crystallography or cryo-EM can identify endpoint structures (Shoemaker and Ando, 2018; Venien-Bryan et al., 2017). NMR can determine whether those structures interconvert in solution, whether ligand binding shifts their populations, and which residues sense the perturbation. Molecular simulations can propose pathways or networks of coupling (Karplus and McCammon, 2002; Shaw et al., 2010). HDX-MS can identify regions with altered protection (Konermann et al., 2011; Mayne, 2016). Mutagenesis and functional assays can test whether residues in the proposed network are required for regulation (Huang et al., 1996; Leach et al., 2018). Enzyme kinetics can determine whether altered dynamics or populations affect catalysis (Henzler-Wildman and Kern, 2007; Dotas et al., 2020; Eisenmesser et al., 2005). No single method fully defines allostery; the mechanism emerges from the integration of structure, dynamics, energetics, and function.

A useful conceptual shift is to view NMR not only as a reporter of allosteric structural changes but as a reporter of allosteric free-energy redistribution. When a ligand binds, it can stabilize one conformation, destabilize another, alter the entropy of fluctuations, reshape transition barriers, or change the coupling between local interactions. NMR observables provide experimental access to these changes through shifts, relaxation rates, exchange parameters, and perturbation correlations. In this sense, NMR can distinguish among mechanisms that may appear similar in static structures: induced fit *versus* conformational selection, structural *versus* dynamic allostery, local pathway *versus* distributed network, and enthalpic stabilization *versus* entropic redistribution.

A central interpretive limitation is that correlated NMR responses do not by themselves establish a causal pathway of allosteric communication. Chemical-shift changes, covariance networks, or altered dynamics can arise from a shared population shift rather than direct residue-to-residue transmission. Perturbation series, functional measurements, and orthogonal structural or computational analyses are therefore needed to distinguish correlation from mechanistic coupling.

For non-specialists, the key point is that allostery is not always visible as a large structural change. For NMR specialists, the opportunity is to design perturbation series and experiments that distinguish changes in structure, dynamics, population, and kinetics. The most powerful allostery studies are not simply maps of chemical shift changes; they are quantitative analyses of how perturbations reshape the conformational and energetic landscape of the molecule.

## 7 What does disorder do?

Intrinsically disordered proteins and regions challenge the idea that biological function requires a stable three-dimensional fold (Wright and Dyson, 1999). Disordered regions can mediate signaling (Wright and Dyson, 2015), scaffolding (Cortese et al., 2008), transcriptional regulation (Wright and Dyson, 2015), autoinhibition (Fenton et al., 2023; Trudeau et al., 2013), degradation (van der Lee et al., 2014), phase separation (Martin and Holehouse, 2020), and multivalent interactions (Fenton et al., 2023; Sipko et al., 2024). They often contain short linear motifs, transient secondary structure, weak long-range contacts, and post-translational modification sites. Their function is encoded not in a single structure but in an ensemble of conformations and interactions.

Solution NMR is one of the primary tools for studying disorder at residue-level resolution (Dyson and Wright, 2021; Camacho-Zarco et al., 2022; Pre et al., 2018). Disordered proteins produce narrow resonances because of their rapid motion, making them accessible even when they are large in sequence length. Chemical shifts report on local conformational preferences and transient secondary structure. Relaxation measurements probe backbone dynamics. PREs detect transient long-range contacts and compaction. Diffusion measurements report on hydrodynamic properties. Hydrogen exchange and solvent accessibility experiments provide information on local protection. Direct carbon detection can be useful for proline-rich or highly disordered systems where conventional amide-based experiments are limited (Cook et al., 2018; Felli and Pierattelli, 2022; Kosol et al., 2013).

The important questions for intrinsically disordered systems are what conformational biases exist, how these biases change upon binding or modification, whether transient contacts organize the ensemble, and how disorder contributes to function. NMR can detect coupled folding and binding, conformational selection within disordered ensembles, fuzzy complexes in which disorder persists after binding, and local structural propensities that tune interaction specificity.

Integration with other methods is essential because disorder is inherently ensemble-based. SAXS provides global dimensions and compaction (Chavas, 2014; Svergun, 2010; Venditti et al., 2015b; Thompson et al., 2019; Bernado et al., 2007). smFRET provides distance distributions and conformational heterogeneity at the single-molecule level (Ha et al., 1996; Okamoto et al., 2018; Robustelli et al., 2012). Molecular simulations generate ensemble models that can be restrained or reweighted using NMR chemical shifts, PREs, J couplings, and RDCs (Purslow et al., 2018; Clore and Schwieters, 2003; Koukos and Bonvin, 2020; Cavalli et al., 2013). Mass spectrometry can identify modifications and protection patterns (Konermann et al., 2011; Mayne, 2016; Leitner et al., 2020; O'Reilly and Rappsilber, 2018; Hernandez and Robinson, 2007; Mehmood et al., 2015). Cellular assays and mutagenesis determine which sequence features matter for function (Huang et al., 1996; Leach et al., 2018; Fowler and Fields, 2014). Together, these approaches can connect sequence, ensemble properties, molecular interactions, and biological output.

For non-specialists, the important message is that disordered does not mean unstructured in a functional sense. Disordered regions can contain organized statistical preferences that are measurable and biologically meaningful. For NMR specialists, the challenge is to avoid overinterpreting sparse observables as single structures and instead use NMR data to define ensembles with appropriate uncertainty. In disorder biology, NMR is most powerful when it is used as an ensemble constraint rather than as a route to a unique model.

## 8 How do large assemblies and molecular machines behave?

Large complexes are often viewed as challenging for solution NMR because slow tumbling leads to broad lines and reduced sensitivity. This limitation is real, but it should not obscure the important role that NMR can play in studying large assemblies. In many biological machines, the most important NMR-accessible information is not the complete structure of the entire assembly, but the behavior of selected sites, domains, ligands, or dynamic regions within it.

Modern labeling and detection strategies have greatly expanded the size range of solution NMR. Perdeuteration, TROSY-based detection, methyl-specific labeling, selective amino-acid labeling, segmental labeling, and fluorine probes allow targeted observation of large proteins and complexes (Abramov et al., 2020; Arntson and Pomerantz, 2016; Gardner and Kay, 1998; Pervushin et al., 1997; Salzmann et al., 1998; Skrisovska et al., 2010; Tugarinov et al., 2006; Tugarinov et al., 2003b; Werle et al., 2025). Methyl-TROSY is particularly powerful because methyl groups in Ile, Leu, Val, Met, Ala, or Thr residues can serve as sensitive probes of side-chain packing, dynamics, ligand binding, and allosteric changes in systems far larger than those accessible by conventional backbone NMR. Fluorine NMR provides sensitive, background-free probes that can be introduced through labels or fluorinated amino acids.

In large assemblies, NMR often answers focused questions. Which domains remain flexible? Which residues contact a binding partner? Does a ligand stabilize one conformational state? Does a disordered tail remain mobile after assembly? Does a subunit exchange between free and bound forms? Are there low-population bound states? DEST and related saturation-transfer methods can detect exchange between visible monomeric states and large invisible assemblies (Anthis and Clore, 2015). PREs can identify transient contacts (Iwahara and Clore, 2006; Tang et al., 2006; Clore and Iwahara, 2009). Methyl chemical shifts and relaxation can report on conformational changes within otherwise inaccessible complexes (Palmer, 2004; Palmer and Koss, 2019).

Integration with cryo-EM is particularly powerful. Cryo-EM can provide the architecture of a large assembly (Fernandez-Leiro and Scheres, 2016), while NMR can identify flexible regions, conformational exchange, ligand-dependent perturbations, and solution behavior (Palmer, 2004; Palmer and Koss, 2019). XL-MS can define proximity restraints (Leitner et al., 2020; O'Reilly and Rappsilber, 2018). Native mass spectrometry can determine stoichiometry (Barth and Schmidt, 2020). SAXS/SANS can describe global organization and conformational heterogeneity (Chavas, 2014; Svergun, 2010). Simulations can model dynamics within the structural framework (Karplus and McCammon, 2002; Shaw et al., 2010). NMR then provides local experimental tests of which parts of the assembly are rigid, dynamic, bound, or exchanging.

The most important conceptual point is that NMR does not need to observe everything in a large assembly to be useful. Indeed, its selectivity is often an advantage. By designing labels and experiments around the functional question, NMR can observe the sites that matter most: an active-site methyl group, a regulatory domain, a disordered region, a ligand, a nucleotide, or a conformational sensor. In integrated structural biology, partial but precise information can be more valuable than incomplete global visibility. This selectivity also imposes an interpretive limitation, because conclusions depend on whether the chosen probes adequately report on the functional process of interest.

## 9 How do membranes, surfaces, and interfaces regulate function?

Many biomolecular processes occur at interfaces. Proteins bind membranes, nucleic-acid surfaces, nanoparticles, cytoskeletal assemblies, organelle surfaces, and other molecular scaffolds. Enzymes act on membrane-associated substrates. Signaling proteins are recruited to lipid bilayers. Disordered regions interact with surfaces through weak multivalent contacts. Catalytic reactions at solid-liquid interfaces can be controlled by transient adsorption and desorption. These interfacial states are often low-population, heterogeneous, and dynamic, making them difficult to characterize with static structural methods.

Solution NMR can detect and quantify such interfacial interactions, especially when the bound state is transient or invisible (An et al., 2022; Anthis and Clore, 2015). CSPs and line broadening can identify residues affected by membrane or surface binding (Zuiderweg, 2002; Purslow et al., 2020; Williamson, 2013; Thompson et al., 2019). PREs from spin-labeled lipids (Clore and Iwahara, 2009), soluble paramagnetic probes (Bernini et al., 2009; Hocking et al., 2013), or surface-attached labels can map proximity to interfaces (Clore and Iwahara, 2009). Diffusion measurements can detect changes in hydrodynamic behavior (Wilkins et al., 1999). Transferred NOE experiments can characterize ligands bound to large receptors or surfaces (Post, 2003). DEST and exchange saturation transfer can detect exchange between a visible free state and a broadened surface-bound state (Fawzi et al., 2011; Fawzi et al., 2012). Fluorine NMR can provide sensitive probes of binding and conformational changes in complex environments (Danielson and Falke, 1996).

These approaches are especially valuable when the bound state is functionally important but difficult to observe directly. A protein may spend only a small fraction of time bound to a membrane, yet that state may be essential for signaling. A small molecule may transiently adsorb to a nanoparticle surface, and its residence time may control catalytic selectivity. A disordered region may weakly contact a surface through many rapidly exchanging interactions. NMR can detect these low-population states through their effects on the visible solution resonances.

Integrated workflows are again essential. Cryo-EM and crystallography may provide structures of stable membrane proteins or complexes (Shoemaker and Ando, 2018; Venien-Bryan et al., 2017). Solid-state NMR can characterize less mobile membrane-associated states (Hong et al., 2012; McDermott, 2009). EPR and fluorescence can report on distances, orientation, and membrane insertion (Dos Santos Rodrigues et al., 2023; Hubbel et al., 2013; Liu et al., 2015). Atomic Force Microscopy (AFM) and microscopy can define surface organization (Al-Amoudi et al., 2004; Muller and Dufrene, 2011; Sartori et al., 2007). Molecular simulations can model membrane binding, adsorption pathways, and residence times (Karplus and McCammon, 2002). Kinetic assays can determine whether interfacial binding affects function (Schuck, 1997). Solution NMR contributes site-specific information on exchange, binding surfaces, and transient states.

For non-specialists, the key point is that NMR can study interactions that are not stable enough to isolate but are still mechanistically important. For NMR specialists, the challenge is to design experiments that distinguish specific binding, nonspecific surface effects, aggregation, viscosity changes, and exchange broadening. When carefully controlled, solution NMR provides a rare window into the dynamic molecular layer between free solution and stable interfacial association.

## 10 How do biomolecular condensates encode molecular interactions and material properties?

Biomolecular condensates formed by liquid-liquid phase separation (LLPS) have become a major focus of modern biology. These assemblies concentrate proteins, nucleic acids, and other biomolecules into membraneless compartments with distinct compositions and material properties (Shin and Brangwynne, 2017; Alberti et al., 2019; Banani et al., 2017). Their formation is often driven by intrinsically disordered regions, folded domains, multivalent interactions, electrostatics, aromatic contacts, cation-pi interactions, nucleic-acid binding, and post-translational modifications (Banani et al., 2017; Borcherds et al., 2021; Li et al., 2012). Condensates can be dynamic and liquid-like, but they can also mature, gel, age, or form more solid-like states (Jawerth et al., 2020; Patel et al., 2015).

A central challenge is to connect molecular interactions to mesoscale properties. Microscopy can reveal droplets, fusion, coarsening, and cellular localization (Banani et al., 2017; Brangwynne et al., 2009; Taylor et al., 2019). Fluorescence recovery after photobleaching (FRAP) can measure molecular recovery (Banani et al., 2017; Brangwynne et al., 2009; Taylor et al., 2019). Rheology can characterize viscoelasticity (Alshareedah et al., 2021; Michieletto and Marenda, 2022). SAXS and SANS can provide information on internal organization and component-specific contrast (Ankner et al., 2013; Martin et al., 2020; Martin et al., 2021; Peran and Mittag, 2020). Simulations can model interaction networks and phase behavior (Dignon et al., 2020; Dignon et al., 2018). However, these approaches often do not directly identify which residues contact each other inside or around condensates. This is where NMR can make a distinctive contribution: solution NMR has been used to connect residue-specific interactions, structure, and dynamics with phase behavior (Murthy and Fawzi, 2020; Bramham and Golovanov, 2022).

Solution NMR can probe the dilute phase, the dense phase when sufficiently mobile, and exchange between observable and unobservable states (Burke et al., 2015; Murthy and Fawzi, 2020; Emmanouilidis et al., 2021; Guseva et al., 2023; Murthy et al., 2019). Residue-specific intensity losses can identify regions incorporated into large assemblies or experiencing enhanced relaxation, while CSPs can report on changes in local environment upon phase separation or client binding. Studies of FUS have used NMR together with complementary measurements to reveal compaction, altered dynamics, and residue-specific interactions associated with condensation (Emmanouilidis et al., 2021; Guseva et al., 2023; Murthy et al., 2019). Fast picosecond-to-nanosecond internal motions of observable resonances can be characterized with R_1_, R_2_, and heteronuclear NOE measurements (Burke et al., 2015; Guseva et al., 2023), whereas off-resonance R_1ρ_ relaxation dispersion reports on slower conformational exchange and has been applied to phase-separated protein systems (Yuwen et al., 2018). PRE measurements require an introduced paramagnetic center, such as a site-specific spin label or paramagnetic metal-based probe; the resulting distance-dependent relaxation enhancements report on intra- or intermolecular proximity (Iwahara et al., 2004; Venditti and Fawzi, 2018). Intermolecular PREs have, for example, been used to map residue-specific contacts within the condensed phase of the FUS low-complexity domain (Murthy et al., 2019). DEST and related saturation-transfer methods can detect exchange between an NMR-visible state and a slowly tumbling or otherwise NMR-invisible associated state (Anthis and Clore, 2015). In a related high-concentration regime, ^19^F DEST has been used to characterize reversible formation of protein-specific large clusters (Edwards et al., 2019). Intermolecular NOEs and relaxation measurements have also identified residue-specific interaction hot spots within condensed phases (Kim et al., 2021). Pulsed-field-gradient diffusion measurements report on translational diffusion and, under suitable conditions, can help characterize changes in molecular mobility associated with partitioning between condensed and dilute phases (Novakovic et al., 2025). Methyl NMR and selective-labeling strategies extend residue-specific measurements to folded domains within condensates (Ahmed et al., 2024).

The most important questions are mechanistic. Which residues drive phase separation? Are contacts specific or distributed? Do folded domains retain structure inside condensates? Do nucleic acids compete with or reorganize protein-protein contacts? How do post-translational modifications alter contact networks and material properties? How does the dense phase differ from the dilute phase? How do client molecules partition and move within condensates? NMR can answer these questions at residue-level resolution when combined with orthogonal measurements of phase behavior and material state.

The integrated nature of condensate biology is unavoidable. NMR identifies contacts and dynamics. Microscopy defines morphology and time evolution. FRAP and rheology define material behavior. SAXS/SANS provide structural information across larger length scales and, in favorable cases, component-specific contrast. Simulations generate molecular ensembles and interaction networks that can be tested against NMR observables. Mutagenesis and phase diagrams determine which interactions are necessary and sufficient for condensation (Das et al., 2020; Wang et al., 2018). The goal is not merely to state that a protein phase separates, but to explain how molecular contacts generate material properties and biological function.

For non-specialists, the key message is that NMR can connect the molecular and material descriptions of condensates. For NMR specialists, condensates require careful attention to sample heterogeneity, exchange, relaxation losses, concentration differences between phases, and the possibility that the most important species may be partially or fully invisible. The disappearance of an NMR signal is not a failure; when interpreted quantitatively, it can be a measurement of incorporation, exchange, or immobilization. Signal loss is therefore potentially informative, but it is not mechanistically unique: incorporation into a condensed phase, intermediate-timescale exchange, increased viscosity, aggregation, and immobilization can produce similar effects and must be distinguished experimentally.

## 11 How can NMR and molecular simulations be combined quantitatively?

Molecular simulations and NMR are natural partners. NMR provides experimental observables that are often ensemble-averaged in space and time. Simulations provide atomistic trajectories that can explain those observables in terms of structures, motions, interactions, and transitions. Neither approach is sufficient alone. NMR data are rich but indirect. Simulations are detailed but model-dependent. Integrated analysis can use the strengths of each to compensate for the limitations of the other.

A central principle is that NMR observables should be compared to simulations through forward models. Chemical shifts (Robustelli et al., 2012), scalar couplings (Schmid and Meuwly, 2008), RDCs (Sedinkin et al., 2023; Robustelli et al., 2018), PREs (Clore and Iwahara, 2009), PCS (Iwahara et al., 2004), NOEs (Cavalli et al., 2013; Cullen et al., 2026), relaxation rates (Lindorff-Larsen et al., 2012), order parameters (Best and Vendruscolo, 2004), hydrogen-exchange protection factors (Best and Vendruscolo, 2006), and diffusion-related observables (Singer et al., 2017) can often be calculated or approximated from structural ensembles. Agreement between calculated and experimental observables supports a model; disagreement identifies missing states, incorrect populations, force-field limitations, or inadequate sampling.

NMR can be used in several simulation workflows. In restrained simulations, NMR observables are included as restraints to bias sampling toward experimentally consistent conformations. In ensemble refinement, a pool of structures is selected or weighted to reproduce experimental data. In maximum entropy or Bayesian reweighting, simulations are minimally adjusted to match observables while preserving as much of the original ensemble as possible. In enhanced sampling, simulations generate rare states or transitions that can be tested by relaxation dispersion, CEST, PREs, or other NMR data. In Markov state models, NMR exchange parameters can help validate state populations and transition kinetics.

This integration is particularly powerful for systems with conformational heterogeneity. A simulation may suggest that a protein samples open and closed states, but NMR can determine whether both states are populated in solution and on what timescale. PREs may reveal transient compaction not captured by an initial simulation (Iwahara and Clore, 2006; Clore and Iwahara, 2009; B et al., 2005). Relaxation dispersion may identify a low-population state that simulations can then attempt to model structurally (Bouvignies et al., 2011). Chemical shifts may indicate local secondary-structure propensities in a disordered ensemble (Jensen et al., 2010). RDCs may constrain domain orientations (Clore et al., 1998b). Thus, NMR does not merely validate simulations globally; it identifies which molecular features of a simulation are experimentally credible.

For non-specialists, the key point is that simulations become more reliable when tested against experimental observables. For NMR specialists, the challenge is to recognize that NMR data rarely define a unique ensemble by themselves. The combination of NMR and simulation is most rigorous when uncertainty is explicit, observables are forward-calculated, and models are penalized for overfitting sparse data. The future of integrated structural biology will increasingly depend on such quantitative frameworks, particularly as AI-generated structural models and large-scale simulations become routine starting points for biological interpretation.

## 12 How does NMR connect structure to biological mechanism?

The ultimate goal of structural biology is not simply to describe molecules but to explain how they work. A structural model becomes mechanistic when it accounts for activity, regulation, specificity, signaling, catalysis, localization, or assembly. Solution NMR contributes to mechanism by connecting molecular observables to functional states and transitions.

In enzymes, NMR can reveal whether ligand binding shifts conformational equilibria, whether catalytic loops exchange between open and closed states, whether mutations alter dynamics without changing the average structure, and whether substrates, products, or inhibitors stabilize distinct conformations. Real-time NMR can monitor reactions directly in favorable cases. Relaxation dispersion can detect conformational states that are populated only transiently but may be catalytically relevant. CSPs can map substrate and cofactor binding. Methyl NMR can monitor active-site packing and domain rearrangements in large enzymes. These data can be integrated with enzyme kinetics, mutagenesis, crystallography, cryo-EM, and simulations to build models linking dynamics to catalysis.

In signaling and regulation, NMR can determine whether post-translational modifications alter local structure, long-range allosteric coupling, disorder, binding affinity, or conformational exchange. In protein-protein interaction networks, NMR can identify weak contacts and conditional recognition surfaces. In nucleic-acid biology, NMR can characterize dynamic RNA structures, protein-RNA interactions, base-pair opening, and ligand-induced conformational changes. In membrane and condensate biology, NMR can identify transient molecular contacts that regulate localization, assembly, and material behavior.

A question-centered framework is essential because mechanism is rarely reducible to a single observable. A ligand-induced chemical shift change may reflect direct binding, an allosteric structural change, altered dynamics, or a population shift. A broadened resonance may indicate exchange, aggregation, surface binding, or intermediate timescale motion. A PRE may reflect a stable contact or a low-population transient encounter. Interpretation requires integration with orthogonal data and with perturbations that test causality. Mutations, ligand analogs, concentration dependence, temperature dependence, kinetic assays, and simulations can help distinguish alternative models.

Thus, the most powerful mechanistic NMR studies are perturbation-based. They ask how the molecular system responds to changes in ligand, mutation, modification, pH, salt, temperature, pressure, concentration, phase state, or binding partner. These perturbations transform NMR spectra from descriptive fingerprints into quantitative maps of molecular response. When combined with functional measurements, they reveal which structural and dynamic changes matter for biological output.

## 13 Practical guide: matching biological questions to NMR strategies

**FIGURE 4:**
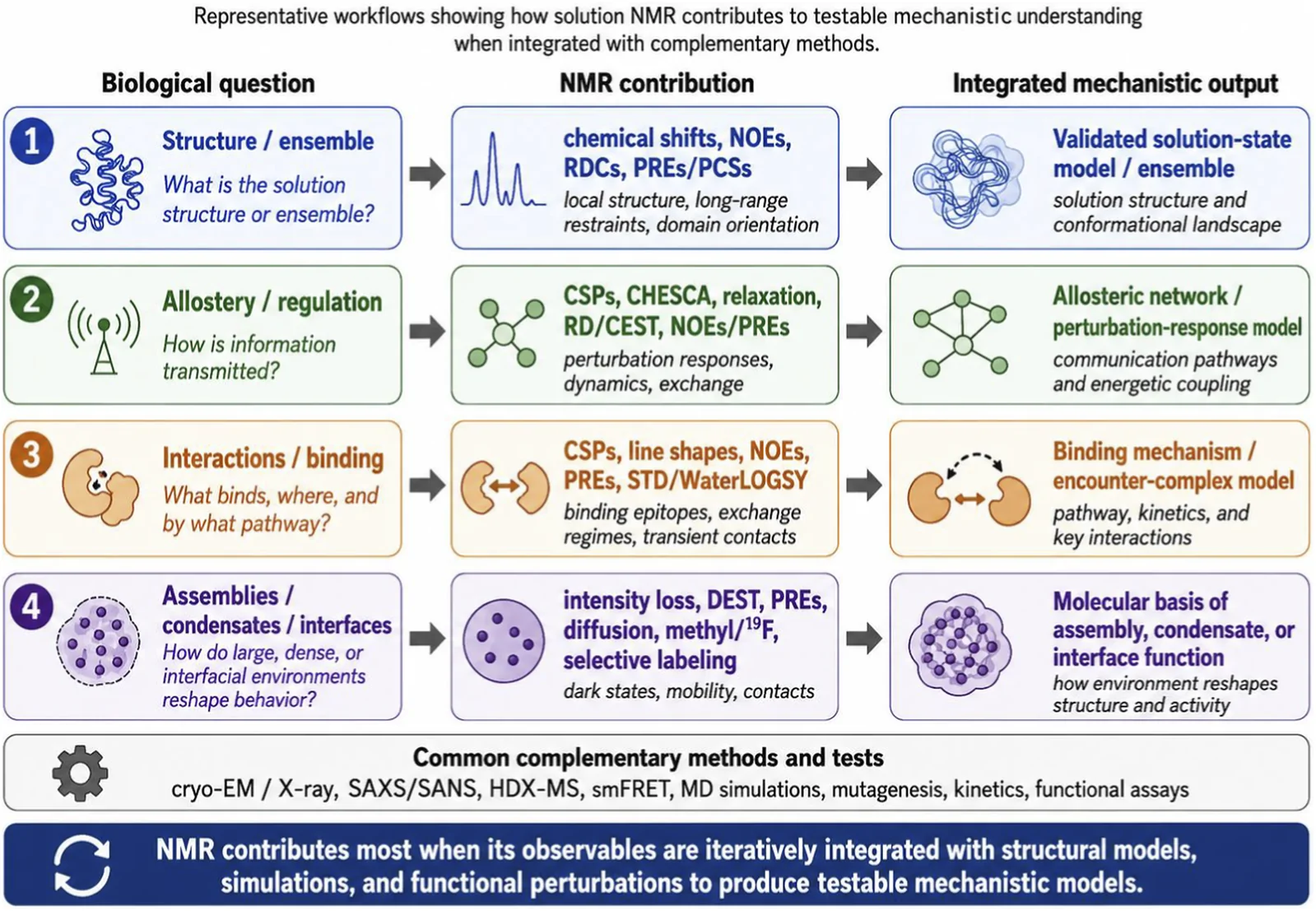
From NMR observables to integrated mechanistic models. Representative workflows show how solution NMR addresses four recurring biological questions: solution structure and ensembles, allostery and regulation, molecular interactions, and assemblies, condensates, or interfaces. For each question, the figure links representative NMR observables to the mechanistic information they provide. Chemical shifts, NOEs, RDCs, PREs, and PCSs constrain solution-state models; CSPs, relaxation, relaxation dispersion, CEST, NOEs, and PREs probe allosteric responses; CSPs, line shapes, NOEs, PREs, and ligand-observed NMR define binding mechanisms; and intensity changes, DEST, PREs, diffusion, and selective labeling probe dark states, mobility, and contacts in large or dense environments. Complementary structural, biophysical, computational, and functional approaches are used to validate and refine the resulting mechanistic models.

The question-centered logic summarized above can be translated into integrated workflows in which NMR observables are combined with structural models, simulations, orthogonal biophysical measurements, and functional perturbations to generate mechanistic models (Figure 4). A complementary practical summary is provided in Table 2, which matches common biological questions with NMR contributions and complementary methods.

**TABLE 2:** Question-centered guide to matching solution NMR approaches with biological questions and complementary structural biology methods.

Biological question	What NMR contributes	NMR approaches	Best integrated with
What is the structure in solution?	Local restraints, domain orientation, flexible regions	NOE, RDC, PRE, PCS, chemical shifts	X-ray, cryo-EM, predicted structures, SAXS, MD
What states are populated?	Populations, exchange rates, excited states	CPMG, R_1ρ_, CEST, DEST, EXSY, line-shape analysis	MD, smFRET, SAXS, HDX-MS
What binds and where?	Binding sites, affinities, exchange regime	CSP, titration, NOEs, PRE, STD, WaterLOGSY	ITC, SPR/BLI, cryo-EM, X-ray, docking
How does allostery work?	Long-range perturbations, dynamic coupling	CHESCA, relaxation, RD/CEST, NOEs/PREs, RDCs	MD networks, kinetics, HDX-MS, mutagenesis
What does disorder do?	Residue-level transient structure and dynamics	Chemical shifts, PRE, relaxation, diffusion	SAXS, smFRET, simulations
How do large assemblies behave?	Dynamic/interface-specific information	Methyl-TROSY, selective labeling, DEST, PRE	Cryo-EM, XL-MS, native mass spectrometry
How do condensates work?	Residue-level contacts and mobility	Intensity, relaxation, PRE, DEST, diffusion	Microscopy, FRAP, SANS/SAXS, rheology
How do surfaces/membranes regulate function?	Low-population bound states, orientation, exchange	PRE, DEST, diffusion,^19^F NMR	EPR, MD, microscopy, kinetics
How can simulations be validated by experiment?	Experimental restraints and ensemble averages	Chemical shifts, PRE, RDC, relaxation	MD, enhanced sampling, ensemble refinement
How is function regulated?	Coupling among binding, dynamics, catalysis, assembly	Real-time NMR, relaxation dispersion, titrations	Kinetics, mutagenesis, cellular assays

These workflows are not intended as rigid recipes. Each system imposes constraints related to size, solubility, concentration, labeling feasibility, exchange regime, and biological context. Nevertheless, these examples emphasize a central point: NMR experiment selection should follow from the biological question and from the missing information in the integrated model.

## 14 Outlook: the future of solution NMR in integrated structural biology

The future of solution NMR will not be defined by a return to the era in which NMR, crystallography, and cryo-EM were viewed as competing structure-determination methods. Instead, NMR will be most influential when it provides quantitative information that other methods cannot easily supply. This includes conformational populations, exchange rates, weak and transient interactions, site-specific dynamics, disorder, hidden states, allosteric coupling, and the molecular basis of phase behavior.

Several developments will strengthen this role. Advances in isotope labeling, methyl NMR, fluorine NMR, nonuniform sampling, sensitivity enhancement, high-field magnets, cryogenic probes, and automated analysis continue to expand the range of systems accessible to solution NMR. Improved integration with cryo-EM will allow atomic models of large assemblies to be complemented by solution dynamics and state populations. Integration with AI-based structure prediction will shift many NMR applications toward model validation, ensemble refinement, and functional interpretation. Better forward models and Bayesian approaches will improve the rigor of NMR-guided simulations. In-cell and *in situ* NMR methods may increasingly connect purified-component mechanisms to cellular environments, although sensitivity, complexity, and interpretation remain challenging.

The value of NMR should not be measured only by the number of complete structures it determines. Instead, its value lies in the mechanistic information it contributes to integrated models. A single residue-specific exchange rate can identify a functional excited state. A pattern of CSPs can reveal an allosteric network. A PRE profile can detect a transient encounter complex. A methyl spectrum can report on ligand-dependent conformational selection in a large enzyme. A loss of intensity in a condensate-forming protein can identify the residues incorporated into a dense phase. These measurements may be partial, but they are often decisive.

For NMR specialists, the challenge is to present experiments in a way that is intelligible and useful to the broader structural biology community. For non-specialists, the challenge is to recognize when a biological question requires the dynamic, quantitative, and residue-specific information that solution NMR can provide. Integrated structural biology succeeds when each method contributes its most informative observable. In that landscape, solution NMR is not merely a legacy structure-determination technique. It is a quantitative engine for transforming structural models into mechanistic understanding.

## 15 Closing perspective

The era of integrated structural biology does not diminish the importance of solution NMR; it clarifies its most powerful role. High-resolution structures, computational models, scattering profiles, imaging data, mass spectrometry measurements, and functional assays each provide indispensable but incomplete views of biomolecular systems. Solution NMR fills a critical gap by measuring how molecules behave in solution: how they fluctuate, bind, exchange, assemble, respond to perturbation, and distribute their conformational energy across multiple states. These properties are often the difference between a structural description and a mechanistic explanation.

A question-centered use of NMR is therefore essential. The most important decision is not which experiment is fashionable or technically impressive, but which molecular uncertainty must be resolved. If the uncertainty is structural, NMR can test and refine solution models. If the uncertainty is dynamic, NMR can quantify motions and exchange. If the uncertainty is interaction-based, NMR can map weak, transient, and heterogeneous binding events. If the uncertainty is regulatory, NMR can reveal how perturbations redistribute structure, dynamics, and populations across a biomolecule. When integrated with orthogonal methods, these observables convert static or partial models into experimentally constrained mechanisms.

The future impact of solution NMR will depend on how clearly the field communicates this role. For specialists, this means presenting NMR data not only as spectra or parameters, but as answers to biological questions. For non-specialists, it means recognizing that many important mechanisms cannot be inferred from structural models alone. Biomolecules are not only objects with shapes; they are dynamic systems with populations, lifetimes, interactions, and responses. Solution NMR remains one of the few methods capable of measuring these properties with site-specific resolution. In the era of integrated structural biology, that capability is not peripheral but central.

## References

[B1] Abramov G. Velyvis A. Rennella E. Wong L. E. Kay L. E. (2020). A methyl-TROSY approach for NMR studies of high-molecular-weight DNA with application to the nucleosome core particle. Proc. Natl. Acad. Sci. U. S. A. 117 (23), 12836–12846. 10.1073/pnas.2004317117 32457157 PMC7293644

[B2] Adamski W. Salvi N. Maurin D. Magnat J. Milles S. Jensen M. R. (2019). A unified description of intrinsically disordered protein dynamics under physiological conditions using NMR spectroscopy. J. Am. Chem. Soc. 141 (44), 17817–17829. 10.1021/jacs.9b09002 31591893

[B3] Ahmed R. Rangadurai A. K. Ruetz L. Tollinger M. Kreutz C. Kay L. E. (2024). A delayed decoupling methyl-TROSY pulse sequence for atomic resolution studies of folded proteins and RNAs in condensates. J. Magn. Reson 362, 107667. 10.1016/j.jmr.2024.107667 38626504

[B4] Al-Amoudi A. Chang J. J. Leforestier A. McDowall A. Salamin L. M. Norlen L. P. (2004). Cryo-electron microscopy of vitreous sections. EMBO J. 23 (18), 3583–3588. 10.1038/sj.emboj.7600366 15318169 PMC517607

[B5] Alberti S. Gladfelter A. Mittag T. (2019). Considerations and challenges in studying liquid-liquid phase separation and biomolecular condensates. Cell 176 (3), 419–434. 10.1016/j.cell.2018.12.035 30682370 PMC6445271

[B6] Alshareedah I. Kaur T. Banerjee P. R. (2021). Methods for characterizing the material properties of biomolecular condensates. Methods Enzymol. 646, 143–183. 10.1016/bs.mie.2020.06.009 33453924 PMC7849318

[B7] An Y. Sedinkin S. L. Venditti V. (2022). Solution NMR methods for structural and thermodynamic investigation of nanoparticle adsorption equilibria. Nanoscale Adv. 4 (12), 2583–2607. 10.1039/d2na00099g 35769933 PMC9195484

[B8] Ankner J. F. Heller W. T. Herwig K. W. Meilleur F. Myles D. A. (2013). Neutron scattering techniques and applications in structural biology. Curr. Protoc. Protein Sci. 17, 16. 10.1002/0471140864.ps1716s72 23546619

[B9] Anthis N. J. Clore G. M. (2015). Visualizing transient dark states by NMR spectroscopy. Q. Rev. Biophys. 48 (1), 35–116. 10.1017/S0033583514000122 25710841 PMC6276111

[B10] Arntson K. E. Pomerantz W. C. (2016). Protein-observed fluorine NMR: a bioorthogonal approach for small molecule discovery. J. Med. Chem. 59 (11), 5158–5171. 10.1021/acs.jmedchem.5b01447 26599421

[B11] Astore M. A. Pradhan A. S. Thiede E. H. Hanson S. M. (2024). Protein dynamics underlying allosteric regulation. Curr. Opin. Struct. Biol. 84, 102768. 10.1016/j.sbi.2023.102768 38215528

[B12] Bertoncini C. W. Jung Y. S. Fernandez C. O. Hoyer W. Griesinger C. Jovin T. M. (2005). Release of long-range tertiary interactions potentiates aggregation of natively unstructured alpha-synuclein. Proc. Natl. Acad. Sci. U. S. A. 102 (5), 1430–1435. 10.1073/pnas.0407146102 15671169 PMC547830

[B13] Bai X. C. McMullan G. Scheres S. H. (2015). How cryo-EM is revolutionizing structural biology. Trends Biochem. Sci. 40 (1), 49–57. 10.1016/j.tibs.2014.10.005 25544475

[B14] Banani S. F. Lee H. O. Hyman A. A. Rosen M. K. (2017). Biomolecular condensates: organizers of cellular biochemistry. Nat. Rev. Mol. Cell Biol. 18 (5), 285–298. 10.1038/nrm.2017.7 28225081 PMC7434221

[B15] Barth M. Schmidt C. (2020). Native mass spectrometry-A valuable tool in structural biology. J. Mass Spectrom. 55 (10), e4578. 10.1002/jms.4578 32662584

[B16] Bates T. A. Gurmessa S. K. Weinstein J. B. Trank-Greene M. Wrynla X. H. Anastas A. (2025). Biolayer interferometry for measuring the kinetics of protein-protein interactions and nanobody binding. Nat. Protoc. 20 (4), 861–883. 10.1038/s41596-024-01079-8 39572731

[B17] Bax A. (2003). Weak alignment offers new NMR opportunities to study protein structure and dynamics. Protein Sci. 12 (1), 1–16. 10.1110/ps.0233303 12493823 PMC2312400

[B18] Bernado P. Mylonas E. Petoukhov M. V. Blackledge M. Svergun D. I. (2007). Structural characterization of flexible proteins using small-angle X-ray scattering. J. Am. Chem. Soc. 129 (17), 5656–5664. 10.1021/ja069124n 17411046

[B19] Bernini A. Venditti V. Spiga O. Niccolai N. (2009). Probing protein surface accessibility with solvent and paramagnetic molecules. Prog. Nucl. Mag. Res. Sp. 54, 278–289. 10.1016/j.pnmrs.2008.10.003

[B20] Bertini I. Luchinat C. Parigi G. Pierattelli R. (2005). NMR spectroscopy of paramagnetic metalloproteins. Chembiochem 6 (9), 1536–1549. 10.1002/cbic.200500124 16094696

[B21] Best R. B. Vendruscolo M. (2004). Determination of protein structures consistent with NMR order parameters. J. Am. Chem. Soc. 126 (26), 8090–8091. 10.1021/ja0396955 15225030

[B22] Best R. B. Vendruscolo M. (2006). Structural interpretation of hydrogen exchange protection factors in proteins: characterization of the native state fluctuations of CI2. Structure 14 (1), 97–106. 10.1016/j.str.2005.09.012 16407069

[B23] Boehr D. D. McElheny D. Dyson H. J. Wright P. E. (2006). The dynamic energy landscape of dihydrofolate reductase catalysis. Science 313 (5793), 1638–1642. 10.1126/science.1130258 16973882

[B24] Borcherds W. Bremer A. Borgia M. B. Mittag T. (2021). How do intrinsically disordered protein regions encode a driving force for liquid–liquid phase separation? Curr. Opin. Struct. Biol. 67, 41–50. 10.1016/j.sbi.2020.09.004 33069007 PMC8044266

[B25] Bouvignies G. Vallurupalli P. Hansen D. F. Correia B. E. Lange O. Bah A. (2011). Solution structure of a minor and transiently formed state of a T4 lysozyme mutant. Nature 477 (7362), 111–114. 10.1038/nature10349 21857680 PMC3706084

[B26] Bramham J. E. Golovanov A. P. (2022). Temporal and spatial characterisation of protein liquid-liquid phase separation using NMR spectroscopy. Nat. Commun. 13 (1), 1767. 10.1038/s41467-022-29408-z 35365630 PMC8976059

[B27] Brangwynne C. P. Eckmann C. R. Courson D. S. Rybarska A. Hoege C. Gharakhani J. (2009). Germline P granules are liquid droplets that localize by controlled dissolution/condensation. Science 324 (5935), 1729–1732. 10.1126/science.1172046 19460965

[B28] Burke K. A. Janke A. M. Rhine C. L. Fawzi N. L. (2015). Residue-by-Residue view of *in vitro* FUS granules that bind the C-terminal domain of RNA polymerase II. Mol. Cell 60 (2), 231–241. 10.1016/j.molcel.2015.09.006 26455390 PMC4609301

[B29] Burns D. Khatiwada B. Singh A. Purslow J. A. Potoyan D. A. Venditti V. (2024). An alpha-ketoglutarate conformational switch controls iron accessibility, activation, and substrate selection of the human FTO protein. Proc. Natl. Acad. Sci. U. S. A. 121 (25), e2404457121. 10.1073/pnas.2404457121 38865275 PMC11194561

[B30] Camacho-Zarco A. R. Schnapka V. Guseva S. Abyzov A. Adamski W. Milles S. (2022). NMR provides unique insight into the functional dynamics and interactions of intrinsically disordered proteins. Chem. Rev. 122, 9331–9356. 10.1021/acs.chemrev.1c01023 35446534 PMC9136928

[B31] Camilloni C. De Simone A. Vranken W. F. Vendruscolo M. (2012). Determination of secondary structure populations in disordered states of proteins using nuclear magnetic resonance chemical shifts. Biochemistry 51 (11), 2224–2231. 10.1021/bi3001825 22360139

[B32] Cavalli A. Salvatella X. Dobson C. M. Vendruscolo M. (2007). Protein structure determination from NMR chemical shifts. Proc. Natl. Acad. Sci. U. S. A. 104 (23), 9615–9620. 10.1073/pnas.0610313104 17535901 PMC1887584

[B33] Cavalli A. Camilloni C. Vendruscolo M. (2013). Molecular dynamics simulations with replica-averaged structural restraints generate structural ensembles according to the maximum entropy principle. J. Chem. Phys. 138 (9), 094112. 10.1063/1.4793625 23485282

[B34] Cavanagh J. Fairbrother W. J. Palmer A. G. Rance M. Skelton N. J. (2007). Protein NMR Spectroscopy: Principles and Practice.

[B35] Chavas L. M. G. (2014). Small angle X-Ray and neutron scattering from solutions of biological macromolecules. Acta Crystallogr. D. Biol. Crystallogr. 70, 1175–1176. 10.1107/S1399004714005719

[B36] Chen J. Vishweshwaraiah Y. L. Dokholyan N. V. (2022). Design and engineering of allosteric communications in proteins. Curr. Opin. Struct. Biol. 73, 102334. 10.1016/j.sbi.2022.102334 35180676 PMC8957532

[B37] Clore G. M. (2008). Visualizing lowly-populated regions of the free energy landscape of macromolecular complexes by paramagnetic relaxation enhancement. Mol. Biosyst. 4 (11), 1058–1069. 10.1039/b810232e 18931781 PMC2807640

[B38] Clore G. M. Gronenborn A. M. (1998). Determining the structures of large proteins and protein complexes by NMR. Trends Biotechnol. 16 (1), 22–34. 10.1016/S0167-7799(97)01135-9 9470228

[B39] Clore G. M. Iwahara J. (2009). Theory, practice, and applications of paramagnetic relaxation enhancement for the characterization of transient low-population states of biological macromolecules and their complexes. Chem. Rev. 109 (9), 4108–4139. 10.1021/cr900033p 19522502 PMC2825090

[B40] Clore G. M. Schwieters C. D. (2003). Docking of protein-protein complexes on the basis of highly ambiguous intermolecular distance restraints derived from 1H/15N chemical shift mapping and backbone 15N-1H residual dipolar couplings using conjoined rigid body/torsion angle dynamics. J. Am. Chem. Soc. 125 (10), 2902–2912. 10.1021/ja028893d 12617657

[B41] Clore G. M. Schwieters C. D. (2004). How much backbone motion in ubiquitin is required to account for dipolar coupling data measured in multiple alignment media as assessed by independent cross-validation? J. Am. Chem. Soc. 126 (9), 2923–2938. 10.1021/ja0386804 14995210

[B42] Clore G. M. Gronenborn A. M. (1998a). NMR structure determination of proteins and protein complexes larger than 20 kDa. Curr. Opin. Chem. Biol. 2 (5), 564–570. 10.1016/s1367-5931(98)80084-7 9818180

[B43] Clore G. M. Gronenborn A. M. Tjandra N. (1998b). Direct structure refinement against residual dipolar couplings in the presence of rhombicity of unknown magnitude. J. Magn. Reson 131 (1), 159–162. 10.1006/jmre.1997.1345 9533920

[B44] Clore G. M. Tang C. Iwahara J. (2007). Elucidating transient macromolecular interactions using paramagnetic relaxation enhancement. Curr. Opin. Struct. Biol. 17 (5), 603–616. 10.1016/j.sbi.2007.08.013 17913493 PMC2134839

[B45] Cook E. C. Usher G. A. Showalter S. A. (2018). The use of (13)C direct-detect NMR to characterize flexible and disordered proteins. Methods Enzymol. 611, 81–100. 10.1016/bs.mie.2018.08.025 30471706

[B46] Cooper A. Dryden D. T. (1984). Allostery without conformational change. A plausible model. Eur. Biophys. J. 11 (2), 103–109. 10.1007/BF00276625 6544679

[B47] Cornilescu G. Delaglio F. Bax A. (1999). Protein backbone angle restraints from searching a database for chemical shift and sequence homology. J. Biomol. NMR 13 (3), 289–302. 10.1023/a:1008392405740 10212987

[B48] Cortese M. S. Uversky V. N. Dunker A. K. (2008). Intrinsic disorder in scaffold proteins: getting more from less. Prog. Biophys. Mol. Biol. 98 (1), 85–106. 10.1016/j.pbiomolbio.2008.05.007 18619997 PMC2671330

[B49] Cullen M. Biancaniello C. Taskova K. Miletic V. Mercadante D. De Simone A. (2026). Integrating NMR restraints into coarse-grained simulations: toward accurate conformational ensembles of complex protein systems. J. Am. Chem. Soc. 148 (12), 13160–13173. 10.1021/jacs.5c22987 41851975 PMC13047695

[B50] Dalvit C. Fogliatto G. Stewart A. Veronesi M. Stockman B. (2001). WaterLOGSY as a method for primary NMR screening: practical aspects and range of applicability. J. Biomol. NMR 21 (4), 349–359. 10.1023/a:1013302231549 11824754

[B51] Dalvit C. Fagerness P. E. Hadden D. T. Sarver R. W. Stockman B. J. (2003). Fluorine-NMR experiments for high-throughput screening: theoretical aspects, practical considerations, and range of applicability. J. Am. Chem. Soc. 125 (25), 7696–7703. 10.1021/ja034646d 12812511

[B52] Danielson M. A. Falke J. J. (1996). Use of 19F NMR to probe protein structure and conformational changes. Annu. Rev. Biophys. Biomol. Struct. 25, 163–195. 10.1146/annurev.bb.25.060196.001115 8800468 PMC2899692

[B53] Das S. Lin Y. H. Vernon R. M. Forman-Kay J. D. Chan H. S. (2020). Comparative roles of charge, pi, and hydrophobic interactions in sequence-dependent phase separation of intrinsically disordered proteins. Proc. Natl. Acad. Sci. U. S. A. 117 (46), 28795–28805. 10.1073/pnas.2008122117 33139563 PMC7682375

[B54] Dignon G. L. Zheng W. Kim Y. C. Best R. B. Mittal J. (2018). Sequence determinants of protein phase behavior from a coarse-grained model. PLoS Comput. Biol. 14 (1), e1005941. 10.1371/journal.pcbi.1005941 29364893 PMC5798848

[B55] Dignon G. L. Best R. B. Mittal J. (2020). Biomolecular phase separation: from molecular driving forces to macroscopic properties. Annu. Rev. Phys. Chem. 71, 53–75. 10.1146/annurev-physchem-071819-113553 32312191 PMC7469089

[B56] Dos Santos Rodrigues F. H. Delgado G. G. Santana da Costa T. Tasic L. (2023). Applications of fluorescence spectroscopy in protein conformational changes and intermolecular contacts. BBA Adv. 3, 100091. 10.1016/j.bbadva.2023.100091 37207090 PMC10189374

[B57] Dotas R. R. Nguyen T. T. Stewart C. E. Jr. Ghirlando R. Potoyan D. A. Venditti V. (2020). Hybrid thermophilic/mesophilic enzymes reveal a role for conformational disorder in regulation of bacterial enzyme I. J. Mol. Biol. 432 (16), 4481–4498. 10.1016/j.jmb.2020.05.024 32504625 PMC7963438

[B58] Dyson H. J. Wright P. E. (2005). Intrinsically unstructured proteins and their functions. Nat. Rev. Mol. Cell Biol. 6 (3), 197–208. 10.1038/nrm1589 15738986

[B59] Dyson H. J. Wright P. E. (2021). NMR illuminates intrinsic disorder. Curr. Opin. Struct. Biol. 70, 44–52. 10.1016/j.sbi.2021.03.015 33951592 PMC8530845

[B60] Edwards J. M. Bramham J. E. Podmore A. Bishop S. M. van der Walle C. F. Golovanov A. P. (2019). (19)F dark-state exchange saturation transfer NMR reveals reversible formation of protein-specific large clusters in high-concentration protein mixtures. Anal. Chem. 91 (7), 4702–4708. 10.1021/acs.analchem.9b00143 30801173 PMC6492951

[B61] Eisenmesser E. Z. Bosco D. A. Akke M. Kern D. (2002). Enzyme dynamics during catalysis. Science 295 (5559), 1520–1523. 10.1126/science.1066176 11859194

[B62] Eisenmesser E. Z. Millet O. Labeikovsky W. Korzhnev D. M. Wolf-Watz M. Bosco D. A. (2005). Intrinsic dynamics of an enzyme underlies catalysis. Nature 438 (7064), 117–121. 10.1038/nature04105 16267559

[B63] Emmanouilidis L. Esteban-Hofer L. Damberger F. F. de Vries T. Nguyen C. K. X. Ibanez L. F. (2021). NMR and EPR reveal a compaction of the RNA-binding protein FUS upon droplet formation. Nat. Chem. Biol. 17 (5), 608–614. 10.1038/s41589-021-00752-3 33686294 PMC7617049

[B64] Esteban-Martin S. Fenwick R. B. Salvatella X. (2010). Refinement of ensembles describing unstructured proteins using NMR residual dipolar couplings. J. Am. Chem. Soc. 132 (13), 4626–4632. 10.1021/ja906995x 20222664

[B65] Fawzi N. L. Doucleff M. Suh J. Y. Clore G. M. (2010). Mechanistic details of a protein-protein association pathway revealed by paramagnetic relaxation enhancement titration measurements. Proc. Natl. Acad. Sci. U. S. A. 107 (4), 1379–1384. 10.1073/pnas.0909370107 20080627 PMC2824347

[B66] Fawzi N. L. Ying J. Ghirlando R. Torchia D. A. Clore G. M. (2011). Atomic-resolution dynamics on the surface of amyloid-beta protofibrils probed by solution NMR. Nature 480 (7376), 268–272. 10.1038/nature10577 22037310 PMC3237923

[B67] Fawzi N. L. Ying J. Torchia D. A. Clore G. M. (2012). Probing exchange kinetics and atomic resolution dynamics in high-molecular-weight complexes using dark-state exchange saturation transfer NMR spectroscopy. Nat. Protoc. 7 (8), 1523–1533. 10.1038/nprot.2012.077 22814391 PMC3500623

[B68] Felli I. C. Pierattelli R. (2022). (13)C direct detected NMR for challenging systems. Chem. Rev. 122 (10), 9468–9496. 10.1021/acs.chemrev.1c00871 35025504 PMC9136920

[B69] Fenton M. Gregory E. Daughdrill G. (2023). Protein disorder and autoinhibition: the role of multivalency and effective concentration. Curr. Opin. Struct. Biol. 83, 102705. 10.1016/j.sbi.2023.102705 37778184 PMC10841074

[B70] Fernandez-Leiro R. Scheres S. H. (2016). Unravelling biological macromolecules with cryo-electron microscopy. Nature 537 (7620), 339–346. 10.1038/nature19948 27629640 PMC5074357

[B71] Fowler D. M. Fields S. (2014). Deep mutational scanning: a new style of protein science. Nat. Methods 11 (8), 801–807. 10.1038/nmeth.3027 25075907 PMC4410700

[B72] Frederick K. K. Marlow M. S. Valentine K. G. Wand A. J. (2007). Conformational entropy in molecular recognition by proteins. Nature 448 (7151), 325–329. 10.1038/nature05959 17637663 PMC4156320

[B73] Gardner K. H. Kay L. E. (1998). The use of 2H, 13C, 15N multidimensional NMR to study the structure and dynamics of proteins. Annu. Rev. Biophys. Biomol. Struct. 27, 357–406. 10.1146/annurev.biophys.27.1.357 9646872

[B74] Gronenborn A. M. (2022). Small, but powerful and attractive: (19)F in biomolecular NMR. Structure 30 (1), 6–14. 10.1016/j.str.2021.09.009 34995480 PMC8797020

[B75] Grutsch S. Bruschweiler S. Tollinger M. (2016). NMR methods to study dynamic allostery. PLoS Comput. Biol. 12 (3), e1004620. 10.1371/journal.pcbi.1004620 26964042 PMC4786136

[B76] Guo J. Zhou H. X. (2016). Protein allostery and conformational dynamics. Chem. Rev. 116 (11), 6503–6515. 10.1021/acs.chemrev.5b00590 26876046 PMC5011433

[B77] Guseva S. Schnapka V. Adamski W. Maurin D. Ruigrok R. W. H. Salvi N. (2023). Liquid-Liquid phase separation modifies the dynamic properties of intrinsically disordered proteins. J. Am. Chem. Soc. 145 (19), 10548–10563. 10.1021/jacs.2c13647 37146977 PMC10197138

[B78] Ha T. Enderle T. Ogletree D. F. Chemla D. S. Selvin P. R. Weiss S. (1996). Probing the interaction between two single molecules: fluorescence resonance energy transfer between a single donor and a single acceptor. Proc. Natl. Acad. Sci. U. S. A. 93 (13), 6264–6268. 10.1073/pnas.93.13.6264 8692803 PMC39010

[B79] Henzler-Wildman K. Kern D. (2007). Dynamic personalities of proteins. Nature 450 (7172), 964–972. 10.1038/nature06522 18075575

[B80] Hernandez H. Robinson C. V. (2007). Determining the stoichiometry and interactions of macromolecular assemblies from mass spectrometry. Nat. Protoc. 2 (3), 715–726. 10.1038/nprot.2007.73 17406634

[B81] Hocking H. G. Zangger K. Madl T. (2013). Studying the structure and dynamics of biomolecules by using soluble paramagnetic probes. Chemphyschem 14 (13), 3082–3094. 10.1002/cphc.201300219 23836693 PMC4171756

[B82] Hong M. Zhang Y. Hu F. (2012). Membrane protein structure and dynamics from NMR spectroscopy. Annu. Rev. Phys. Chem. 63, 1–24. 10.1146/annurev-physchem-032511-143731 22136620 PMC4082981

[B83] Huang B. Eberstadt M. Olejniczak E. T. Meadows R. P. Fesik S. W. (1996). NMR structure and mutagenesis of the Fas (APO-1/CD95) death domain. Nature 384 (6610), 638–641. 10.1038/384638a0 8967952

[B84] Huang Y. Reddy K. D. Bracken C. Qiu B. Zhan W. Eliezer D. (2023). Environmentally ultrasensitive fluorine probe to resolve protein conformational ensembles by (19)F NMR and Cryo-EM. J. Am. Chem. Soc. 145 (15), 8583–8592. 10.1021/jacs.3c01003 37023263 PMC10119980

[B85] Hubbell W. L. Lopez C. J. Altenbach C. Yang Z. (2013). Technological advances in site-directed spin labeling of proteins. Curr. Opin. Struct. Biol. 23 (5), 725–733. 10.1016/j.sbi.2013.06.008 23850140 PMC3805720

[B86] Hus J. C. Marion D. Blackledge M. (2001). Determination of protein backbone structure using only residual dipolar couplings. J. Am. Chem. Soc. 123 (7), 1541–1542. 10.1021/ja005590f 11456746

[B87] Hyman A. A. Weber C. A. Julicher F. (2014). Liquid-liquid phase separation in biology. Annu. Rev. Cell Dev. Biol. 30, 39–58. 10.1146/annurev-cellbio-100913-013325 25288112

[B88] Iwahara J. Clore G. M. (2006). Detecting transient intermediates in macromolecular binding by paramagnetic NMR. Nature 440 (7088), 1227–1230. 10.1038/nature04673 16642002

[B89] Iwahara J. Schwieters C. D. Clore G. M. (2004). Ensemble approach for NMR structure refinement against (1)H paramagnetic relaxation enhancement data arising from a flexible paramagnetic group attached to a macromolecule. J. Am. Chem. Soc. 126 (18), 5879–5896. 10.1021/ja031580d 15125681

[B90] Iwahara J. Tang C. Marius Clore G. (2007). Practical aspects of (1)H transverse paramagnetic relaxation enhancement measurements on macromolecules. J. Magn. Reson 184 (2), 185–195. 10.1016/j.jmr.2006.10.003 17084097 PMC1994582

[B91] Jawerth L. Fischer-Friedrich E. Saha S. Wang J. Franzmann T. Zhang X. (2020). Protein condensates as aging Maxwell fluids. Science 370 (6522), 1317–1323. 10.1126/science.aaw4951 33303613

[B92] Jensen M. R. Salmon L. Nodet G. Blackledge M. (2010). Defining conformational ensembles of intrinsically disordered and partially folded proteins directly from chemical shifts. J. Am. Chem. Soc. 132 (4), 1270–1272. 10.1021/ja909973n 20063887

[B93] Johnson C. M. (2021). Isothermal titration calorimetry. Methods Mol. Biol. 2263, 135–159. 10.1007/978-1-0716-1197-5_5 33877596

[B94] Jumper J. Evans R. Pritzel A. Green T. Figurnov M. Ronneberger O. (2021). Highly accurate protein structure prediction with AlphaFold. Nature 596 (7873), 583–589. 10.1038/s41586-021-03819-2 34265844 PMC8371605

[B95] Karplus M. McCammon J. A. (2002). Molecular dynamics simulations of biomolecules. Nat. Struct. Biol. 9 (9), 646–652. 10.1038/nsb0902-646 12198485

[B96] Kay L. E. (1998). Protein dynamics from NMR. Biochem. Cell Biol. 76 (2-3), 145–152. 10.1139/bcb-76-2-3-145 9923683

[B97] Kendrew J. C. Bodo G. Dintzis H. M. Parrish R. G. Wyckoff H. Phillips D. C. (1958). A three-dimensional model of the myoglobin molecule obtained by x-ray analysis. Nature 181 (4610), 662–666. 10.1038/181662a0 13517261

[B98] Kern D. Zuiderweg E. R. (2003). The role of dynamics in allosteric regulation. Curr. Opin. Struct. Biol. 13 (6), 748–757. 10.1016/j.sbi.2003.10.008 14675554

[B99] Kim T. H. Payliss B. J. Nosella M. L. Lee I. T. W. Toyama Y. Forman-Kay J. D. (2021). Interaction hot spots for phase separation revealed by NMR studies of a CAPRIN1 condensed phase. Proc. Natl. Acad. Sci. U. S. A. 118 (23), e2104897118. 10.1073/pnas.2104897118 34074792 PMC8201762

[B100] Konermann L. Pan J. Liu Y. H. (2011). Hydrogen exchange mass spectrometry for studying protein structure and dynamics. Chem. Soc. Rev. 40 (3), 1224–1234. 10.1039/c0cs00113a 21173980

[B101] Kosol S. Contreras-Martos S. Cedeno C. Tompa P. (2013). Structural characterization of intrinsically disordered proteins by NMR spectroscopy. Molecules 18 (9), 10802–10828. 10.3390/molecules180910802 24008243 PMC6269831

[B102] Koukos P. I. Bonvin A. (2020). Integrative modelling of biomolecular complexes. J. Mol. Biol. 432 (9), 2861–2881. 10.1016/j.jmb.2019.11.009 31783069

[B103] Kriwacki R. W. Hengst L. Tennant L. Reed S. I. Wright P. E. (1996). Structural studies of p21Waf1/Cip1/Sdi1 in the free and Cdk2-bound state: conformational disorder mediates binding diversity. Proc. Natl. Acad. Sci. U. S. A. 93 (21), 11504–11509. 10.1073/pnas.93.21.11504 8876165 PMC38087

[B104] Kuhlbrandt W. (2014). Biochemistry. The resolution revolution. Science 343 (6178), 1443–1444. 10.1126/science.1251652 24675944

[B105] Leach B. I. Zhang X. Kelly J. W. Dyson H. J. Wright P. E. (2018). NMR measurements reveal the structural basis of transthyretin destabilization by pathogenic mutations. Biochemistry 57 (30), 4421–4430. 10.1021/acs.biochem.8b00642 29972637 PMC6067956

[B106] Leitner A. Bonvin A. Borchers C. H. Chalkley R. J. Chamot-Rooke J. Combe C. W. (2020). Toward increased reliability, transparency, and accessibility in cross-linking mass spectrometry. Structure 28 (11), 1259–1268. 10.1016/j.str.2020.09.011 33065067

[B107] Li P. Banjade S. Cheng H. C. Kim S. Chen B. Guo L. (2012). Phase transitions in the assembly of multivalent signalling proteins. Nature 483 (7389), 336–340. 10.1038/nature10879 22398450 PMC3343696

[B108] Lindorff-Larsen K. Trbovic N. Maragakis P. Piana S. Shaw D. E. (2012). Structure and dynamics of an unfolded protein examined by molecular dynamics simulation. J. Am. Chem. Soc. 134 (8), 3787–3791. 10.1021/ja209931w 22339051

[B109] Lippincott-Schwartz J. Snapp E. Kenworthy A. (2001). Studying protein dynamics in living cells. Nat. Rev. Mol. Cell Biol. 2 (6), 444–456. 10.1038/35073068 11389468

[B110] Liu L. Mayo D. J. Sahu I. D. Zhou A. Zhang R. McCarrick R. M. (2015). Determining the secondary structure of membrane proteins and peptides *via* Electron Spin Echo Envelope Modulation (ESEEM) spectroscopy. Methods Enzymol. 564, 289–313. 10.1016/bs.mie.2015.06.037 26477255 PMC4814931

[B111] Long D. Bouvignies G. Kay L. E. (2014). Measuring hydrogen exchange rates in invisible protein excited states. Proc. Natl. Acad. Sci. U. S. A. 111 (24), 8820–8825. 10.1073/pnas.1405011111 24889628 PMC4066525

[B112] Martin E. W. Holehouse A. S. (2020). Intrinsically disordered protein regions and phase separation: sequence determinants of assembly or lack thereof. Emerg. Top. Life Sci. 4 (3), 307–329. 10.1042/ETLS20190164 33078839

[B113] Martin E. W. Holehouse A. S. Peran I. Farag M. Incicco J. J. Bremer A. (2020). Valence and patterning of aromatic residues determine the phase behavior of prion-like domains. Science 367 (6478), 694–699. 10.1126/science.aaw8653 32029630 PMC7297187

[B114] Martin E. W. Hopkins J. B. Mittag T. (2021). Small-angle X-ray scattering experiments of monodisperse intrinsically disordered protein samples close to the solubility limit. Methods Enzymol. 646, 185–222. 10.1016/bs.mie.2020.07.002 33453925 PMC8370720

[B115] Mayer M. Meyer B. (1999). Characterization of ligand binding by saturation transfer difference NMR spectroscopy. Angew. Chem. Int. Ed. Engl. 38 (12), 1784–1788. 10.1002/(SICI)1521-3773(19990614)38:12<1784::AID-ANIE1784>3.0.CO;2-Q 29711196

[B116] Mayne L. (2016). Hydrogen exchange mass spectrometry. Methods Enzymol. 566, 335–356. 10.1016/bs.mie.2015.06.035 26791986 PMC5858910

[B117] McDermott A. (2009). Structure and dynamics of membrane proteins by magic angle spinning solid-state NMR. Annu. Rev. Biophys. 38, 385–403. 10.1146/annurev.biophys.050708.133719 19245337

[B118] Mehmood S. Allison T. M. Robinson C. V. (2015). Mass spectrometry of protein complexes: from origins to applications. Annu. Rev. Phys. Chem. 66, 453–474. 10.1146/annurev-physchem-040214-121732 25594852

[B119] Michieletto D. Marenda M. (2022). Rheology and viscoelasticity of proteins and nucleic acids condensates. JACS Au 2 (7), 1506–1521. 10.1021/jacsau.2c00055 35911447 PMC9326828

[B120] Mittermaier A. Kay L. E. (2006). New tools provide new insights in NMR studies of protein dynamics. Science 312 (5771), 224–228. 10.1126/science.1124964 16614210

[B121] Monod J. Wyman J. Changeux J. P. (1965). On the Nature of allosteric transitions: a plausible model. J. Mol. Biol. 12, 88–118. 10.1016/s0022-2836(65)80285-6 14343300

[B122] Motlagh H. N. Wrabl J. O. Li J. Hilser V. J. (2014). The ensemble nature of allostery. Nature 508 (7496), 331–339. 10.1038/nature13001 24740064 PMC4224315

[B123] Muller D. J. Dufrene Y. F. (2011). Atomic force microscopy: a nanoscopic window on the cell surface. Trends Cell Biol. 21 (8), 461–469. 10.1016/j.tcb.2011.04.008 21664134

[B124] Murthy A. C. Fawzi N. L. (2020). The (un)structural biology of biomolecular liquid-liquid phase separation using NMR spectroscopy. J. Biol. Chem. 295 (8), 2375–2384. 10.1074/jbc.REV119.009847 31911439 PMC7039561

[B125] Murthy A. C. Dignon G. L. Kan Y. Zerze G. H. Parekh S. H. Mittal J. (2019). Molecular interactions underlying liquid-liquid phase separation of the FUS low-complexity domain. Nat. Struct. Mol. Biol. 26 (7), 637–648. 10.1038/s41594-019-0250-x 31270472 PMC6613800

[B126] Nguyen T. T. Venditti V. (2020). An allosteric pocket for inhibition of bacterial Enzyme I identified by NMR-based fragment screening. J. Struct. Biol. X 4, 100034. 10.1016/j.yjsbx.2020.100034 32743545 PMC7385036

[B127] Nguyen T. T. Ghirlando R. Venditti V. (2018). The oligomerization state of bacterial enzyme I (EI) determines EI's allosteric stimulation or competitive inhibition by alpha-ketoglutarate. J. Biol. Chem. 293 (7), 2631–2639. 10.1074/jbc.RA117.001466 29317499 PMC5818175

[B128] Novakovic M. Han Y. Kathe N. C. Ni Y. Emmanouilidis L. Allain F. H. (2025). LLPS REDIFINE allows the biophysical characterization of multicomponent condensates without tags or labels. Nat. Commun. 16 (1), 4628. 10.1038/s41467-025-59759-2 40389460 PMC12089286

[B129] O'Reilly F. J. Rappsilber J. (2018). Cross-linking mass spectrometry: methods and applications in structural, molecular and systems biology. Nat. Struct. Mol. Biol. 25 (11), 1000–1008. 10.1038/s41594-018-0147-0 30374081

[B130] Okamoto K. Hiroshima M. Sako Y. (2018). Single-molecule fluorescence-based analysis of protein conformation, interaction, and oligomerization in cellular systems. Biophys. Rev. 10 (2), 317–326. 10.1007/s12551-017-0366-3 29243093 PMC5899725

[B131] Oxenfarth A. Kummerer F. Bottaro S. Schnieders R. Pinter G. Jonker H. R. A. (2023). Integrated NMR/Molecular dynamics determination of the ensemble conformation of a thermodynamically stable CUUG RNA tetraloop. J. Am. Chem. Soc. 145 (30), 16557–16572. 10.1021/jacs.3c03578 37479220 PMC10401711

[B132] Palmer A. G. (2004). 3rd, NMR characterization of the dynamics of biomacromolecules. Chem. Rev. 104 (8), 3623–3640. 10.1021/cr030413t 15303831

[B133] Palmer A. G. (2015). 3rd, Enzyme dynamics from NMR spectroscopy. Acc. Chem. Res. 48 (2), 457–465. 10.1021/ar500340a 25574774 PMC4334254

[B134] Palmer A. G. 3rd Koss H. (2019). Chemical exchange. Methods Enzymol. 615, 177–236. 10.1016/bs.mie.2018.09.028 30638530 PMC7493007

[B135] Patel A. Lee H. O. Jawerth L. Maharana S. Jahnel M. Hein M. Y. (2015). A liquid-to-solid phase transition of the ALS protein FUS accelerated by disease mutation. Cell 162 (5), 1066–1077. 10.1016/j.cell.2015.07.047 26317470

[B136] Peran I. Mittag T. (2020). Molecular structure in biomolecular condensates. Curr. Opin. Struct. Biol. 60, 17–26. 10.1016/j.sbi.2019.09.007 31790873 PMC7117980

[B137] Perutz M. F. Rossmann M. G. Cullis A. F. Muirhead H. Will G. North A. C. (1960). Structure of haemoglobin: a three-dimensional Fourier synthesis at 5.5-A. resolution, obtained by X-ray analysis. Nature 185 (4711), 416–422. 10.1038/185416a0 18990801

[B138] Pervushin K. Riek R. Wider G. Wuthrich K. (1997). Attenuated T2 relaxation by mutual cancellation of dipole-dipole coupling and chemical shift anisotropy indicates an avenue to NMR structures of very large biological macromolecules in solution. Proc. Natl. Acad. Sci. U. S. A. 94 (23), 12366–12371. 10.1073/pnas.94.23.12366 9356455 PMC24947

[B139] Pintacuda G. John M. Su X. C. Otting G. (2007). NMR structure determination of protein-ligand complexes by lanthanide labeling. Acc. Chem. Res. 40 (3), 206–212. 10.1021/ar050087z 17370992

[B140] Popovych N. Sun S. Ebright R. H. Kalodimos C. G. (2006). Dynamically driven protein allostery. Nat. Struct. Mol. Biol. 13 (9), 831–838. 10.1038/nsmb1132 16906160 PMC2757644

[B141] Popovych N. Tzeng S. R. Tonelli M. Ebright R. H. Kalodimos C. G. (2009). Structural basis for cAMP-mediated allosteric control of the catabolite activator protein. Proc. Natl. Acad. Sci. U. S. A. 106 (17), 6927–6932. 10.1073/pnas.0900595106 19359484 PMC2678429

[B142] Post C. B. (2003). Exchange-transferred NOE spectroscopy and bound ligand structure determination. Curr. Opin. Struct. Biol. 13 (5), 581–588. 10.1016/j.sbi.2003.09.012 14568612

[B143] Prestel A. Bugge K. Staby L. Hendus-Altenburger R. Kragelund B. B. (2018). Characterization of dynamic IDP complexes by NMR spectroscopy. Methods Enzymol. 611, 193–226. 10.1016/bs.mie.2018.08.026 30471688

[B144] Purslow J. A. Nguyen T. T. Egner T. K. Dotas R. R. Khatiwada B. Venditti V. (2018). Active site breathing of Human Alkbh5 revealed by solution NMR and accelerated molecular dynamics. Biophys. J. 115 (10), 1895–1905. 10.1016/j.bpj.2018.10.004 30352661 PMC6303414

[B145] Purslow J. A. Khatiwada B. Bayro M. J. Venditti V. (2020). NMR methods for structural characterization of protein-protein complexes. Front. Mol. Biosci. 7, 9. 10.3389/fmolb.2020.00009 32047754 PMC6997237

[B146] Purslow J. A. Nguyen T. T. Khatiwada B. Singh A. Venditti V. (2021). N (6)-methyladenosine binding induces a metal-centered rearrangement that activates the human RNA demethylase Alkbh5. Sci. Adv. 7 (34), eabi8215. 10.1126/sciadv.abi8215 PMC8373141 34407931

[B147] Putnam C. D. Hammel M. Hura G. L. Tainer J. A. (2007). X-ray solution scattering (SAXS) combined with crystallography and computation: defining accurate macromolecular structures, conformations and assemblies in solution. Q. Rev. Biophys. 40 (3), 191–285. 10.1017/S0033583507004635 18078545

[B148] Rizuan A. Shenoy J. Mohanty P. Dos Passos P. M. Mercado Ortiz J. F. Bai L. (2025). Structural details of helix-mediated multimerization of the conserved region of TDP-43 C-terminal domain. Nat. Commun. 16 (1), 10528. 10.1038/s41467-025-65546-w 41298366 PMC12658102

[B149] Robustelli P. Stafford K. A. Palmer A. G. (2012). 3rd, Interpreting protein structural dynamics from NMR chemical shifts. J. Am. Chem. Soc. 134 (14), 6365–6374. 10.1021/ja300265w 22381384 PMC3324661

[B150] Robustelli P. Piana S. Shaw D. E. (2018). Developing a molecular dynamics force field for both folded and disordered protein states. Proc. Natl. Acad. Sci. U. S. A. 115 (21), E4758–E4766. 10.1073/pnas.1800690115 29735687 PMC6003505

[B151] Rosenzweig R. Kay L. E. (2014). Bringing dynamic molecular machines into focus by methyl-TROSY NMR. Annu. Rev. Biochem. 83, 291–315. 10.1146/annurev-biochem-060713-035829 24905784

[B152] Saio T. Yokochi M. Kumeta H. Inagaki F. (2010). PCS-based structure determination of protein-protein complexes. J. Biomol. NMR 46 (4), 271–280. 10.1007/s10858-010-9401-4 20300805 PMC2844537

[B153] Salzmann M. Pervushin K. Wider G. Senn H. Wuthrich K. (1998). TROSY in triple-resonance experiments: new perspectives for sequential NMR assignment of large proteins. Proc. Natl. Acad. Sci. U. S. A. 95 (23), 13585–13590. 10.1073/pnas.95.23.13585 9811843 PMC24862

[B154] Sartori A. Gatz R. Beck F. Rigort A. Baumeister W. Plitzko J. M. (2007). Correlative microscopy: bridging the gap between fluorescence light microscopy and cryo-electron tomography. J. Struct. Biol. 160 (2), 135–145. 10.1016/j.jsb.2007.07.011 17884579

[B155] Schmid F. F. Meuwly M. (2008). Direct comparison of experimental and calculated NMR scalar coupling constants for force field validation and adaptation. J. Chem. Theory Comput. 4 (11), 1949–1958. 10.1021/ct800241d 26620337

[B156] Schmitz C. Vernon R. Otting G. Baker D. Huber T. (2012). Protein structure determination from pseudocontact shifts using ROSETTA. J. Mol. Biol. 416 (5), 668–677. 10.1016/j.jmb.2011.12.056 22285518 PMC3638895

[B157] Schuck P. (1997). Use of surface plasmon resonance to probe the equilibrium and dynamic aspects of interactions between biological macromolecules. Annu. Rev. Biophys. Biomol. Struct. 26, 541–566. 10.1146/annurev.biophys.26.1.541 9241429

[B158] Sedinkin S. L. Burns D. Shukla D. Potoyan D. A. Venditti V. (2023). Solution structure ensembles of the open and closed forms of the approximately 130 kDa enzyme I *via* AlphaFold modeling, coarse grained simulations, and NMR. J. Am. Chem. Soc. 145 (24), 13347–13356. 10.1021/jacs.3c03425 37278728 PMC10772991

[B159] Sedinkin S. L. Roche J. Venditti V. (2024). Elucidation of the mechanisms of inter-domain coupling in the monomeric State of enzyme I by high-pressure NMR. J. Mol. Biol. 436 (9), 168553. 10.1016/j.jmb.2024.168553 38548260 PMC11042970

[B160] Selvaratnam R. Chowdhury S. VanSchouwen B. Melacini G. (2011). Mapping allostery through the covariance analysis of NMR chemical shifts. Proc. Natl. Acad. Sci. U. S. A. 108 (15), 6133–6138. 10.1073/pnas.1017311108 21444788 PMC3076865

[B161] Shaw D. E. Maragakis P. Lindorff-Larsen K. Piana S. Dror R. O. Eastwood M. P. (2010). Atomic-level characterization of the structural dynamics of proteins. Science 330 (6002), 341–346. 10.1126/science.1187409 20947758

[B162] Shen Y. Delaglio F. Cornilescu G. Bax A. (2009). TALOS+: a hybrid method for predicting protein backbone torsion angles from NMR chemical shifts. J. Biomol. NMR 44 (4), 213–223. 10.1007/s10858-009-9333-z 19548092 PMC2726990

[B163] Shin Y. Brangwynne C. P. (2017). Liquid phase condensation in cell physiology and disease. Science 357 (6357), eaaf4382. 10.1126/science.aaf4382 28935776

[B164] Shoemaker S. C. Ando N. (2018). X-rays in the cryo-electron microscopy era: structural biology's dynamic future. Biochemistry 57 (3), 277–285. 10.1021/acs.biochem.7b01031 29227642 PMC5999524

[B165] Singer P. M. Asthagiri D. Chapman W. G. Hirasaki G. J. (2017). Molecular dynamics simulations of NMR relaxation and diffusion of bulk hydrocarbons and water. J. Magn. Reson 277, 15–24. 10.1016/j.jmr.2017.02.001 28189994

[B166] Singh A. Purslow J. A. Venditti V. (2021). 15N CPMG relaxation dispersion for the investigation of protein conformational dynamics on the micros-ms timescale. J. Vis. Exp. 170, e62395. 10.3791/62395 PMC8168574 33938889

[B167] Singh A. Pettini F. Gianibbi B. Das S. Barisani D. Purslow J. A. (2026). Structure-based discovery of a non-competitive FTO inhibitor bound to a cryptic site at the domain interface. J. Mol. Biol. 438 (2), 169575. 10.1016/j.jmb.2025.169575 41354147 PMC12709851

[B168] Sipko E. L. Chappell G. F. Berlow R. B. (2024). Multivalency emerges as a common feature of intrinsically disordered protein interactions. Curr. Opin. Struct. Biol. 84, 102742. 10.1016/j.sbi.2023.102742 38096754

[B169] Skeens E. Lisi G. P. (2023). Analysis of coordinated NMR chemical shifts to map allosteric regulatory networks in proteins. Methods 209, 40–47. 10.1016/j.ymeth.2022.12.002 36535575 PMC10173519

[B170] Skrisovska L. Schubert M. Allain F. H. (2010). Recent advances in segmental isotope labeling of proteins: NMR applications to large proteins and glycoproteins. J. Biomol. NMR 46 (1), 51–65. 10.1007/s10858-009-9362-7 19690964

[B171] Svergun D. I. (2010). Small-angle X-ray and neutron scattering as a tool for structural systems biology. Biol. Chem. 391 (7), 737–743. 10.1515/BC.2010.093 20482320

[B172] Tang C. Iwahara J. Clore G. M. (2006). Visualization of transient encounter complexes in protein-protein association. Nature 444 (7117), 383–386. 10.1038/nature05201 17051159

[B173] Tang C. Schwieters C. D. Clore G. M. (2007). Open-to-closed transition in Apo maltose-binding protein observed by paramagnetic NMR. Nature 449 (7165), 1078–1082. 10.1038/nature06232 17960247

[B174] Tanious F. A. Nguyen B. Wilson W. D. (2008). Biosensor-surface plasmon resonance methods for quantitative analysis of biomolecular interactions. Methods Cell Biol. 84, 53–77. 10.1016/S0091-679X(07)84003-9 17964928

[B175] Taylor N. O. Wei M. T. Stone H. A. Brangwynne C. P. (2019). Quantifying dynamics in phase-separated condensates using fluorescence recovery after photobleaching. Biophys. J. 117 (7), 1285–1300. 10.1016/j.bpj.2019.08.030 31540706 PMC6818185

[B176] Thompson R. D. Baisden J. T. Zhang Q. (2019). NMR characterization of RNA small molecule interactions. Methods 167, 66–77. 10.1016/j.ymeth.2019.05.015 31128236 PMC6756962

[B177] Tjandra N. Bax A. (1997). Direct measurement of distances and angles in biomolecules by NMR in a dilute liquid crystalline medium. Science 278 (5340), 1111–1114. 10.1126/science.278.5340.1111 9353189

[B178] Trudeau T. Nassar R. Cumberworth A. Wong E. T. Woollard G. Gsponer J. (2013). Structure and intrinsic disorder in protein autoinhibition. Structure 21 (3), 332–341. 10.1016/j.str.2012.12.013 23375259

[B179] Tugarinov V. Hwang P. M. Ollerenshaw J. E. Kay L. E. (2003a). Cross-correlated relaxation enhanced 1H[bond]13C NMR spectroscopy of methyl groups in very high molecular weight proteins and protein complexes. J. Am. Chem. Soc. 125 (34), 10420–10428. 10.1021/ja030153x 12926967

[B180] Tugarinov V. Kay L. E. Ile L. (2003b). Val methyl assignments of the 723-residue malate synthase G using a new labeling strategy and novel NMR methods. J. Am. Chem. Soc. 125 (45), 13868–13878. 10.1021/ja030345s 14599227

[B181] Tugarinov V. Kay L. E. (2004). An isotope labeling strategy for methyl TROSY spectroscopy. J. Biomol. NMR 28 (2), 165–172. 10.1023/B:JNMR.0000013824.93994.1f 14755160

[B182] Tugarinov V. Kanelis V. Kay L. E. (2006). Isotope labeling strategies for the study of high-molecular-weight proteins by solution NMR spectroscopy. Nat. Protoc. 1 (2), 749–754. 10.1038/nprot.2006.101 17406304

[B183] Tzeng S. R. Kalodimos C. G. (2009). Dynamic activation of an allosteric regulatory protein. Nature 462 (7271), 368–372. 10.1038/nature08560 19924217

[B184] Tzeng S. R. Kalodimos C. G. (2011). Protein dynamics and allostery: an NMR view. Curr. Opin. Struct. Biol. 21 (1), 62–67. 10.1016/j.sbi.2010.10.007 21109422

[B185] Tzeng S. R. Kalodimos C. G. (2012). Protein activity regulation by conformational entropy. Nature 488 (7410), 236–240. 10.1038/nature11271 22801505

[B186] Vallurupalli P. Sekhar A. Yuwen T. Kay L. E. (2017). Probing conformational dynamics in biomolecules *via* chemical exchange saturation transfer: a primer. J. Biomol. NMR 67 (4), 243–271. 10.1007/s10858-017-0099-4 28317074

[B187] van der Lee R. Buljan M. Lang B. Weatheritt R. J. Daughdrill G. W. Dunker A. K. (2014). Classification of intrinsically disordered regions and proteins. Chem. Rev. 114 (13), 6589–6631. 10.1021/cr400525m 24773235 PMC4095912

[B188] Venditti V. Clore G. M. (2012). Conformational selection and substrate binding regulate the monomer/dimer equilibrium of the C-terminal domain of Escherichia coli enzyme I. J. Biol. Chem. 287 (32), 26989–26998. 10.1074/jbc.M112.382291 22722931 PMC3411034

[B189] Venditti V. Fawzi N. L. (2018). Probing the atomic structure of transient protein contacts by paramagnetic relaxation enhancement solution NMR. Methods Mol. Biol. 1688, 243–255. 10.1007/978-1-4939-7386-6_12 29151213 PMC5823026

[B190] Venditti V. Niccolai N. Butcher S. E. (2008). Measuring the dynamic surface accessibility of RNA with the small paramagnetic molecule TEMPOL. Nucleic Acids Res. 36 (4), e20. 10.1093/nar/gkm1062 18056080 PMC2275091

[B191] Venditti V. Clos L. Niccolai N. Butcher S. E. (2009). Minimum-energy path for a u6 RNA conformational change involving protonation, base-pair rearrangement and base flipping. J. Mol. Biol. 391 (5), 894–905. 10.1016/j.jmb.2009.07.003 19591840 PMC2799254

[B192] Venditti V. Tugarinov V. Schwieters C. D. Grishaev A. Clore G. M. (2015a). Large interdomain rearrangement triggered by suppression of micro-to millisecond dynamics in bacterial Enzyme I. Nat. Commun. 6, 5960. 10.1038/ncomms6960 25581904 PMC4293084

[B193] Venditti V. Schwieters C. D. Grishaev A. Clore G. M. (2015b). Dynamic equilibrium between closed and partially closed states of the bacterial Enzyme I unveiled by solution NMR and X-ray scattering. Proc. Natl. Acad. Sci. U. S. A. 112 (37), 11565–11570. 10.1073/pnas.1515366112 26305976 PMC4577164

[B194] Venditti V. Egner T. K. Clore G. M. (2016). Hybrid approaches to structural characterization of conformational ensembles of complex macromolecular systems combining NMR residual dipolar couplings and solution X-ray scattering. Chem. Rev. 116 (11), 6305–6322. 10.1021/acs.chemrev.5b00592 26739383 PMC5590664

[B195] Venien-Bryan C. Li Z. Vuillard L. Boutin J. A. (2017). Cryo-electron microscopy and X-ray crystallography: complementary approaches to structural biology and drug discovery. Acta Crystallogr. F. Struct. Biol. Commun. 73 (Pt 4), 174–183. 10.1107/S2053230X17003740 28368275 PMC5379166

[B196] Wang J. Choi J. M. Holehouse A. S. Lee H. O. Zhang X. Jahnel M. (2018). A molecular grammar governing the driving forces for phase separation of prion-like RNA binding proteins. Cell 174 (3), 688–699 e16. 10.1016/j.cell.2018.06.006 29961577 PMC6063760

[B197] Ward A. B. Sali A. Wilson I. A. (2013). Biochemistry. Integrative structural biology. Science 339 (6122), 913–915. 10.1126/science.1228565 23430643 PMC3633482

[B198] Waudby C. A. Ramos A. Cabrita L. D. Christodoulou J. (2016). Two-dimensional NMR lineshape analysis. Sci. Rep. 6, 24826. 10.1038/srep24826 27109776 PMC4843008

[B199] Werle Y. Kovermann M. (2025). Fluorine labeling and (19)F NMR spectroscopy to study biological molecules and molecular complexes. Chemistry 31 (2), e202402820. 10.1002/chem.202402820 39466678

[B200] Wilkins D. K. Grimshaw S. B. Receveur V. Dobson C. M. Jones J. A. Smith L. J. (1999). Hydrodynamic radii of native and denatured proteins measured by pulse field gradient NMR techniques. Biochemistry 38 (50), 16424–16431. 10.1021/bi991765q 10600103

[B201] Williamson M. P. (2013). Using chemical shift perturbation to characterise ligand binding. Prog. Nucl. Magn. Reson Spectrosc. 73, 1–16. 10.1016/j.pnmrs.2013.02.001 23962882

[B202] Wishart D. S. Sykes B. D. (1994). The 13C chemical-shift index: a simple method for the identification of protein secondary structure using 13C chemical-shift data. J. Biomol. NMR 4 (2), 171–180. 10.1007/BF00175245 8019132

[B203] Wodak S. J. Paci E. Dokholyan N. V. Berezovsky I. N. Horovitz A. Li J. (2019). Allostery in its many disguises: from theory to applications. Structure 27 (4), 566–578. 10.1016/j.str.2019.01.003 30744993 PMC6688844

[B204] Wright P. E. Dyson H. J. (1999). Intrinsically unstructured proteins: re-assessing the protein structure-function paradigm. J. Mol. Biol. 293 (2), 321–331. 10.1006/jmbi.1999.3110 10550212

[B205] Wright P. E. Dyson H. J. (2015). Intrinsically disordered proteins in cellular signalling and regulation. Nat. Rev. Mol. Cell Biol. 16 (1), 18–29. 10.1038/nrm3920 25531225 PMC4405151

[B206] Wuthrich K. (2001). The way to NMR structures of proteins. Nat. Struct. Biol. 8 (11), 923–925. 10.1038/nsb1101-923 11685234

[B207] Wuthrich K. (2003). NMR studies of structure and function of biological macromolecules (Nobel lecture). Angew. Chem. Int. Ed. Engl. 42 (29), 3340–3363. 10.1002/anie.200300595 12888958

[B208] Yuwen T. Brady J. P. Kay L. E. (2018). Probing conformational exchange in weakly interacting, slowly exchanging protein systems *via* off-resonance R(1rho) experiments: application to studies of protein phase separation. J. Am. Chem. Soc. 140 (6), 2115–2126. 10.1021/jacs.7b09576 29303268

[B209] Zuiderweg E. R. (2002). Mapping protein-protein interactions in solution by NMR spectroscopy. Biochemistry 41 (1), 1–7. 10.1021/bi011870b 11771996

[B210] Zweckstetter M. (2021). Introduction: biophysics of biomolecular condensates. Protein Sci. 30 (7), 1274–1276. 10.1002/pro.4135 34085339 PMC8197424

